# Vimentin knockout results in increased expression of sub-endothelial basement membrane components and carotid stiffness in mice

**DOI:** 10.1038/s41598-017-12024-z

**Published:** 2017-09-14

**Authors:** Benoit Langlois, Ekaterina Belozertseva, Ara Parlakian, Mustapha Bourhim, Jacqueline Gao-Li, Jocelyne Blanc, Lei Tian, Dario Coletti, Carlos Labat, Zhor Ramdame-Cherif, Pascal Challande, Véronique Regnault, Patrick Lacolley, Zhenlin Li

**Affiliations:** 1 Inserm, UMR_S 1116, Université de Lorraine, Nancy, France; 2 Sorbonne Universités, UPMC Univ Paris 06, INSERM ERL U1164, Institut Biologie Paris-Seine, Paris, CNRS, UMR 8256 France; 3 Sorbonne Universités, UPMC Univ Paris 06, Institut Jean Le Rond d’Alembert, Paris, CNRS, UMR 7190 France

## Abstract

Intermediate filaments are involved in stress-related cell mechanical properties and in plasticity via the regulation of focal adhesions (FAs) and the actomyosin network. We investigated whether vimentin regulates endothelial cells (ECs) and vascular smooth muscle cells (SMCs) and thereby influences vasomotor tone and arterial stiffness. Vimentin knockout mice (Vim^−/−^) exhibited increased expression of laminin, fibronectin, perlecan, collagen IV and VE-cadherin as well as von Willebrand factor deposition in the subendothelial basement membrane. Smooth muscle (SM) myosin heavy chain, α-SM actin and smoothelin were decreased in Vim^−/−^ mice. Electron microscopy revealed a denser endothelial basement membrane and increased SM cell-matrix interactions. Integrin α_v_, talin and vinculin present in FAs were increased in Vim^−/−^ mice. Phosphorylated FA kinase and its targets Src and ERK1/2 were elevated in Vim^−/−^ mice. Knockout of vimentin, but not of synemin, resulted in increased carotid stiffness and contractility and endothelial dysfunction, independently of blood pressure and the collagen/elastin ratio. The increase in arterial stiffness in Vim^−/−^ mice likely involves vasomotor tone and endothelial basement membrane organization changes. At the tissue level, the results show the implication of FAs both in ECs and vascular SMCs in the role of vimentin in arterial stiffening.

## Introduction

Intermediate filaments (IF), actin-containing microfilaments and microtubules are the three main cytoskeletal systems involved in the transfer of mechanical forces from the cell membrane to the nucleus. The IF family contains more than 70 genes that provide a versatile, tunable, self-assembled network that is interconnected strongly with the other filament types^1–4^. IF proteins are anchored at focal adhesions (FAs) to mediate integrin mechano-transduction in response to extracellular matrix stiffness^5–7^.

Vimentin (Vim), a 57 kDa type III IF protein which is found in precursor neural and mesenchymal cells during mouse embryo development, is replaced progressively by tissue-specific IF members, such as the muscle-specific IF protein, desmin (Des), in muscle cells. In adult mice, Vim is expressed mainly in mesenchyme-derived cells including fibroblasts, endothelial cells (ECs) and vascular smooth muscle cells (SMCs). There exists a gradient in the Vim/Des ratio in the vascular tree of humans and mice^8,9^. Higher Vim content is found in larger arteries such as the aorta and the carotid artery while Des-positive cells are predominant in small-sized muscular arteries such as the mesenteric artery. A third member of IF family, synemin (Synm) co-assembles with different IF partners, in particular Des and Vim, and participates in the dynamics of FAs via its interactions with talin, vinculin and zyxin^10–15^. Vim is a component of FAs^16–19^ where it binds directly or indirectly to integrins via structures termed vimentin associated matrix adhesions (VAMs)^18,20,21^.

The main extracellular matrix components, vasomotor tone and vascular SMC-matrix interactions are thought to be major determinants of arterial stiffness^22–24^. It has been reported also that intrinsic stiffness of vascular SMCs is increased in association with increased aortic stiffness^24–26^. Recently, increased stiffness and adhesion properties of vascular SMCs in hypertension have been shown to accelerate age-related aortic stiffness^27^. Activation of the serum response factor (SRF)/myocardin transcription pathway is responsible for increased SMC stiffness and thereby plays a central role in hypertension-mediated aortic stiffening^28,29^. Using an inducible SMC-specific knockout mouse model of the SRF gene, we have demonstrated that SRF-related decreases in vasomotor tone and cell-matrix attachment in SMCs decreased arterial stiffness in large arteries^30^. Endothelial mechanisms have been proposed to be involved in stiffening of ECs due to aberrant endothelial signaling and subsequent reduction in production and bioavailability of vasoactive factors^27^. The endothelium exerts a regulatory effect on vascular SMC tone via NO release and shear stress^31^. The age-related increase in arterial stiffness is also due to the loss of endothelial regulation of vascular SMC proliferation and to the production of reactive oxygen species^32^.

In Des knockout mice, vascular SMCs have lost a part of their connections to the extracellular matrix, and carotid arteries from Des^−/−^ mice had higher vascular stiffness and arterial wall viscosity compared with Des^+/+^ mice, without changes in arterial thickness or in elastin and collagen contents^33^. In contrast to Des, expressed only in SMCs, Vim and Synm are expressed in both SMCs and ECs of the artery. Contrasting reports show that Vim decreases FA size on the one hand^5,34^ and increases cell stiffness on the other^35,36^ which raises the hypothesis that Vim and/or Synm exert a complex regulating action on SMC and EC functions and are therefore involved in arterial stiffening. In the present study we exploited Vim knockout (Vim^−/−^) and/or Synm knockout (Synm^−/−^) mice to clarify the role of these IF proteins in the mechanical properties of the arterial wall. We further assessed expression of specific markers of ECs and SMCs, and the organization of basement membranes and focal adhesion complexes. We demonstrate a key role for Vim, endothelial basement membrane structure and function, and FAs in arterial stiffness. We have thus identified a targeted approach to treat arterial stiffening.

## Results

### Expression of vimentin in vascular cells

**Figure 1:**
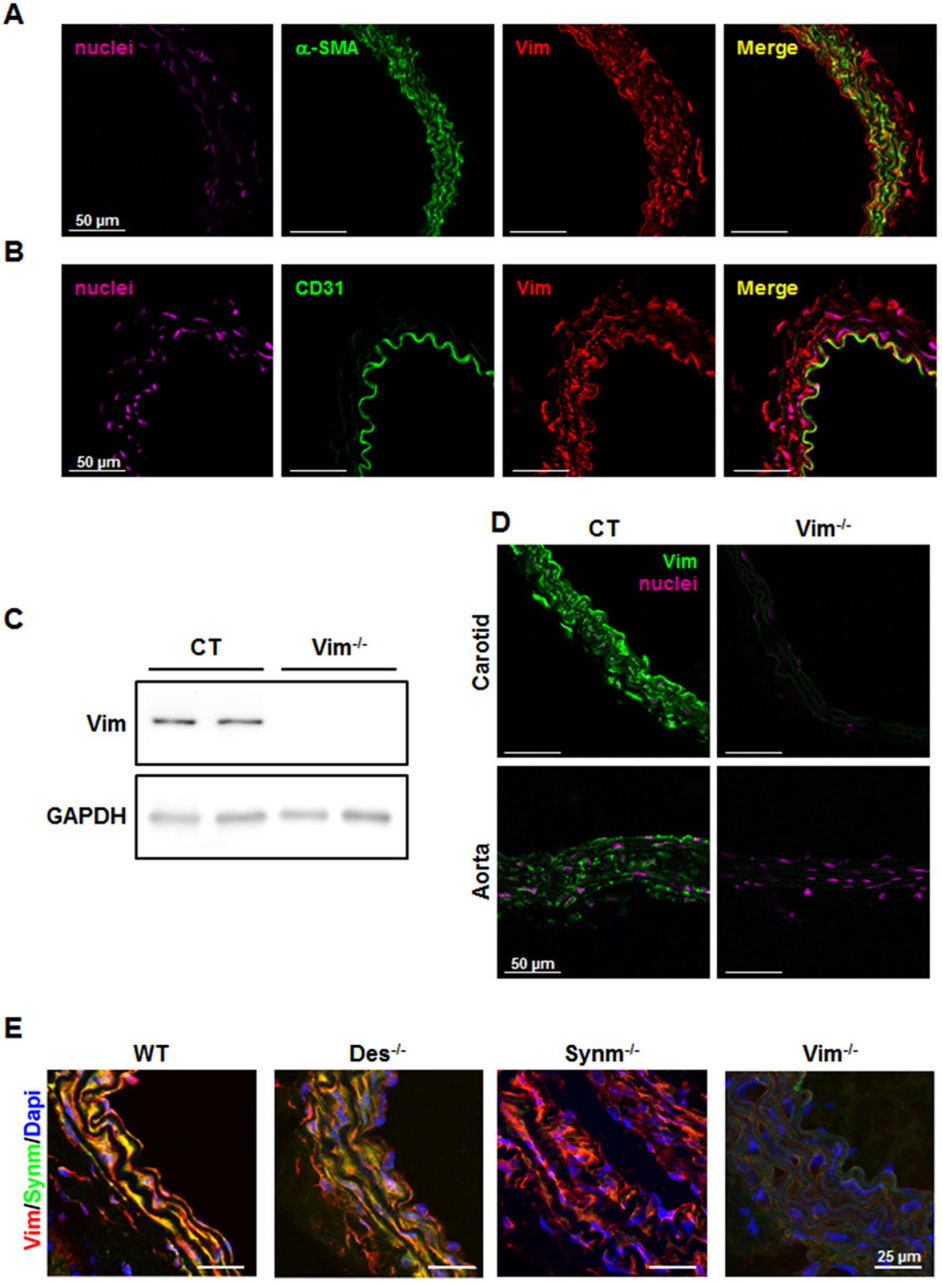
Expression of Vim and its partners in carotid artery and aorta in mice. Confocal immunofluorescence images of the carotid artery shows the presence of Vim (red) in SM-α-actin-positive (green) SMCs (**A**) and CD31-positive (green)ECs (**B**) in CT mice. (**C**) Western blot analysis indicates the knockout of Vim in the carotid artery of Vim^−/−^ mice. (**D**) Immunofluorescence staining shows the knockout of Vim in the carotid artery and aorta of Vim^−/−^ mice. (**E**) Confocal immunofluorescence images show co-localization of Vim (red) and Synm (green) in wild-type (WT) mice and Des^−/−^ mice. Synemin intermediate filaments are absent in Vim^−/−^ mice while Vim filaments are present in Synm^−/−^ mice. Scale bar = 50 μm in panels A, B and D and 25 μm in panel E.

To examine the expression of Vim in arteries, we stained the carotid artery of adult mice with antibodies against α-smooth muscle actin (SMA) in SMCs and to CD31 in ECs. Both α-SMA-positive and CD31-positive cells stained for Vim indicating that Vim was present in SMCs and ECs (Fig. 1A,B). Western blot and immunostaining in Vim^−/−^ mice confirmed total knockout in all Vim-expressing cells (Fig. 1C,D). To investigate whether loss of Vim affected Synm expression, carotid arteries of Vim^−/−^, Synm^−/−^ and Des^−/−^ mice were stained for Vim and Synm. Vim and Synm filaments were present in the carotids of control (CT) mice and Des^−/−^ mice. Whereas positive staining for Vim filaments was observed in Synm^−/−^ mice, Vim^−/−^ arteries showed an absence of Synm (Fig. 1E). Previous studies have shown that the presence of Synm in IF is dependent on the presence of Vim but not of Des in the vessels of the mouse embryo^10^. We confirmed this observation in adult mice.

### Increased expression of components of the basement membrane in Vim^−/−^ mice

**Figure 2:**
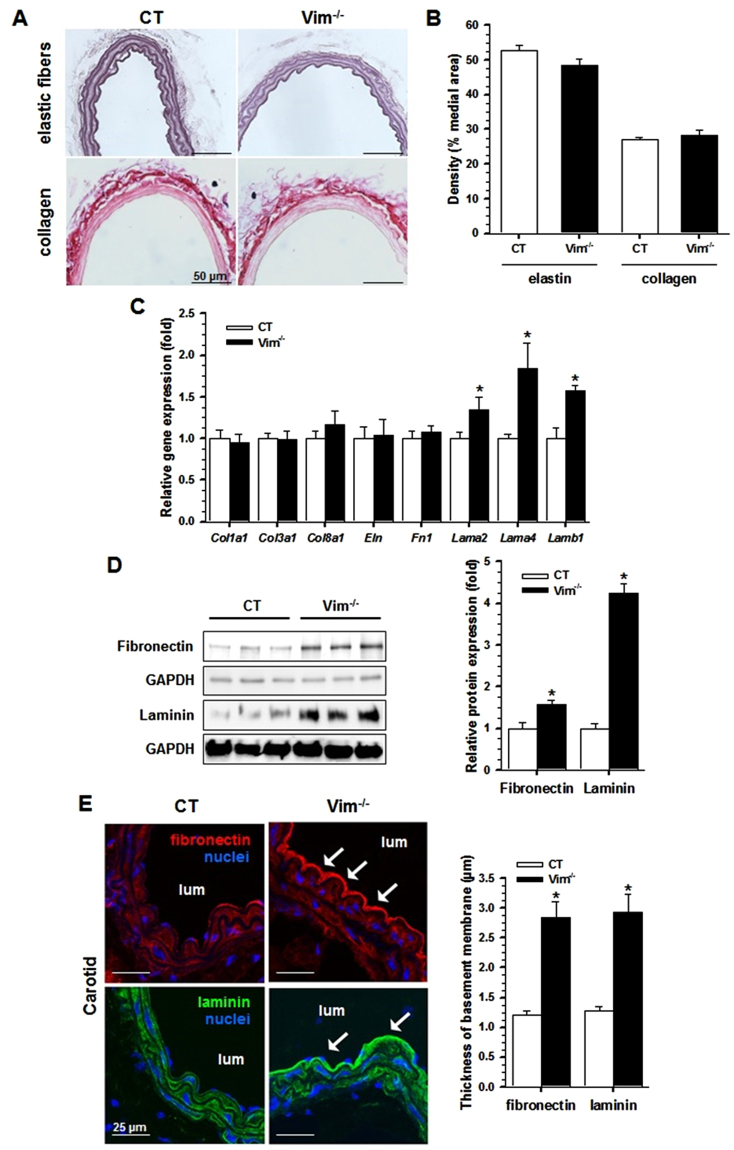
Effect of Vim knockout on matrix proteins in carotid artery. (**A**) Histomorphometry from Vim^−/−^ and CT mice. Cross-sections of carotid artery were stained with Weigert’s resorcin-fuchsin for elastin and Sirius red for collagen. (**B**) Elastin and collagen density in the media. (**C**) Relative mRNA levels (Vim^−/−^ vs CT) of genes encoding several ECM proteins of the carotid artery by qRT-PCR. (**D**) Western blot analysis of fibronectin and laminin in the carotid artery of Vim^−/−^ mice. Results are expressed as means ± SEM (n ≥ 3 in each group). *P < 0.05 compared with CT mice by unpaired Student’s t-test. (**E**) Confocal immunofluorescence images show the increase in fibronectin and laminin in the subendothelial basement membrane (arrows). The thickness of basement membrane after staining by anti-fibronectin and anti-laminin antibodies is measured and expressed as means ± SEM (n = 3 in each group). *P < 0.05 compared with CT mice by unpaired Student’s t-test. Scale bars = 50 µm in panel A and 25 µm in panel E.

**Figure 3:**
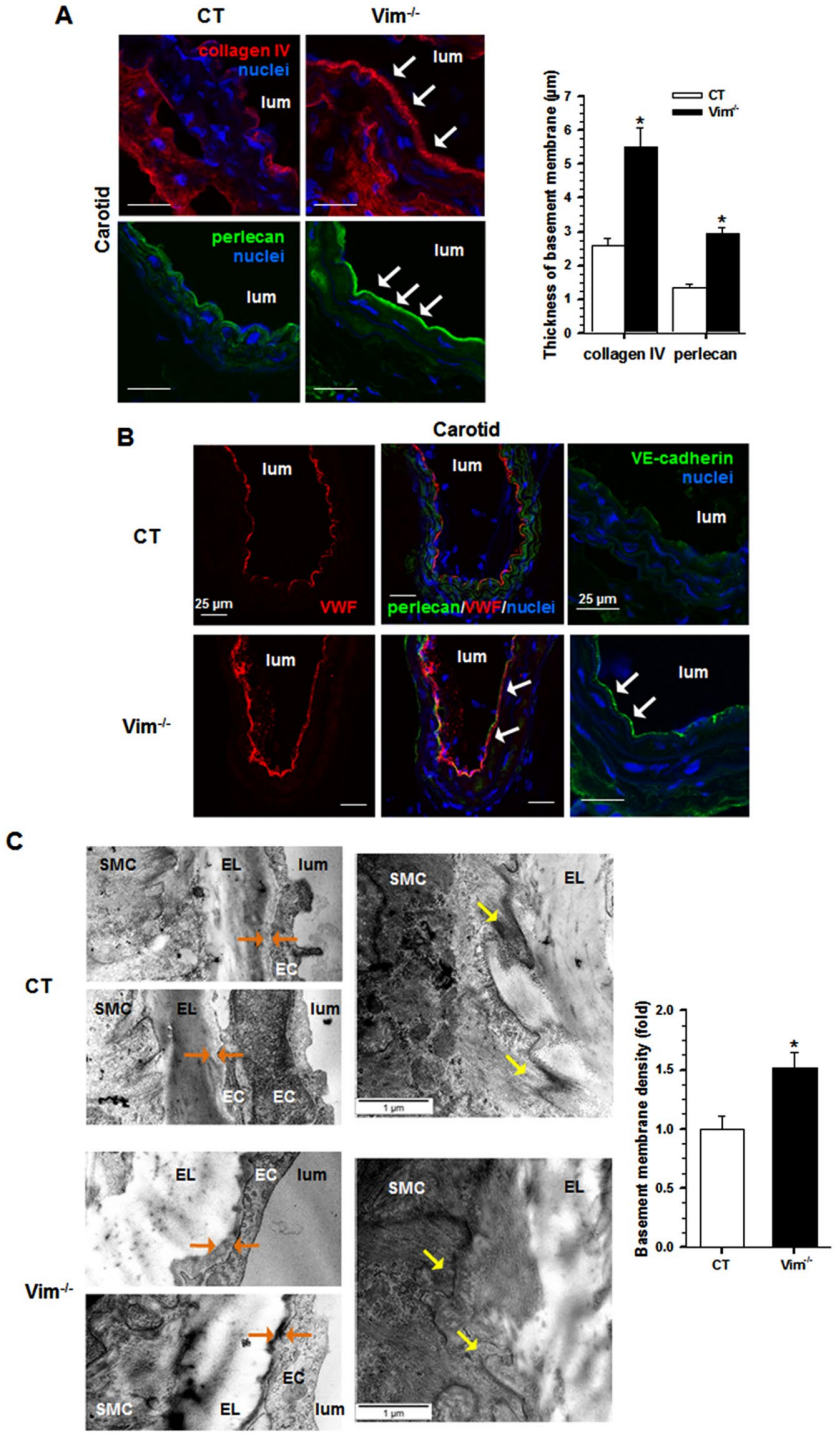
Effect of Vim knockout on the structure of the carotid artery. (**A**) Confocal immunofluorescence images show the increased expression of basement membrane components, collagen IV and perlecan (arrows), in Vim^−/−^ mice. The thickness of the basement membrane after staining by anti-collagen IV and anti-perlecan antibodies is measured and expressed as means ± SEM (n = 3 in each group). *P < 0.05 compared with CT mice by unpaired Student’s t-test. (**B**) Confocal immunofluorescence images show the increased expression (arrows) of the specific endothelial cell markers, von Willebrand factor (VWF) and VE-cadherin, in Vim^−/−^ mice. The arrows indicate deposits of VWF underneath perlecan. (**C**) Electron microscopic analysis from Vim^−/−^ and CT mice. The basement membrane of ECs contains more dense materials (orange arrows) in Vim^−/−^ mice. Yellow arrows indicate less finger-like projections in SMCs from Vim^−/−^ mice. The average basement membrane intensity was measured and is expressed as means ± SEM (n = 3 in each group). *P < 0.05 compared with CT mice by unpaired Student’s t-test. Scale bars = 25 µm in panels A and B and 1 µm in panel C.

To investigate whether Vim knockout affects the micromechanical environment of ECs, we examined the vascular wall and the specific components of the basement membrane in CT and Vim^−/−^ carotid walls using histomorphometry. In Vim^−/−^ mice, there were no changes in carotid elastin and collagen densities compared with CT mice (Fig. 2A,B). The loss of Vim increased mRNA levels of *Lama2*, *Lama4* and *Lamb1* genes, whose products are basement membrane components, (Fig. 2C). There were no changes in the expression of the matrix genes *Col1a1*, *Col3a1*, *Col8a1*, *Eln* and *Fn1*. The total levels of fibronectin and laminin were increased in Vim^−/−^ mice (Fig. 2D). Immunofluorescence staining analysis showed that these increases occurred predominantly in the subendothelial basement membrane (Fig. 2E). We saw also a marked increase in perlecan and collagen IV in the subendothelium (Fig. 3A). In Des^−/−^ mice, only collagen IV is increased in the subendothelium (Supplementary Fig. S1). The thickness of basement membrane in Vim^−/−^ was twice that in CT mice. To further evaluate the deposition of EC-derived proteins, we stained for specific endothelial markers VE-cadherin and von Willebrand Factor (VWF). Both markers were increased in Vim^−/−^ mice (Fig. 3B). VWF staining differed in that deposition extended underneath perlecan, a feature not observed in CT mice.

Electron microscopy examination indicated that the basement membrane area between the internal elastic lamina and ECs contained more dense material in Vim^−/−^ mice (Fig. 3C). Using morphometric analysis, we found an increase in 50% of the average basement membrane intensity in Vim^−/−^ mice compared to CT mice. In the latter, the SMCs displayed fingerlike projections to anchor them to the elastic lamellae. In Vim^−/−^ mice, there were less SMC fingerlike projections and they presented a dense smooth membrane profile. Taken together these data demonstrate a markedly altered expression of the components of the basement membrane surrounding ECs and SMCs in Vim^−/−^ mice in the absence of any modification of the major extracellular proteins that are known to contribute to arterial stiffening.

### Decreased expression of SMC differentiation markers in Vim^−/−^ mice

**Figure 4:**
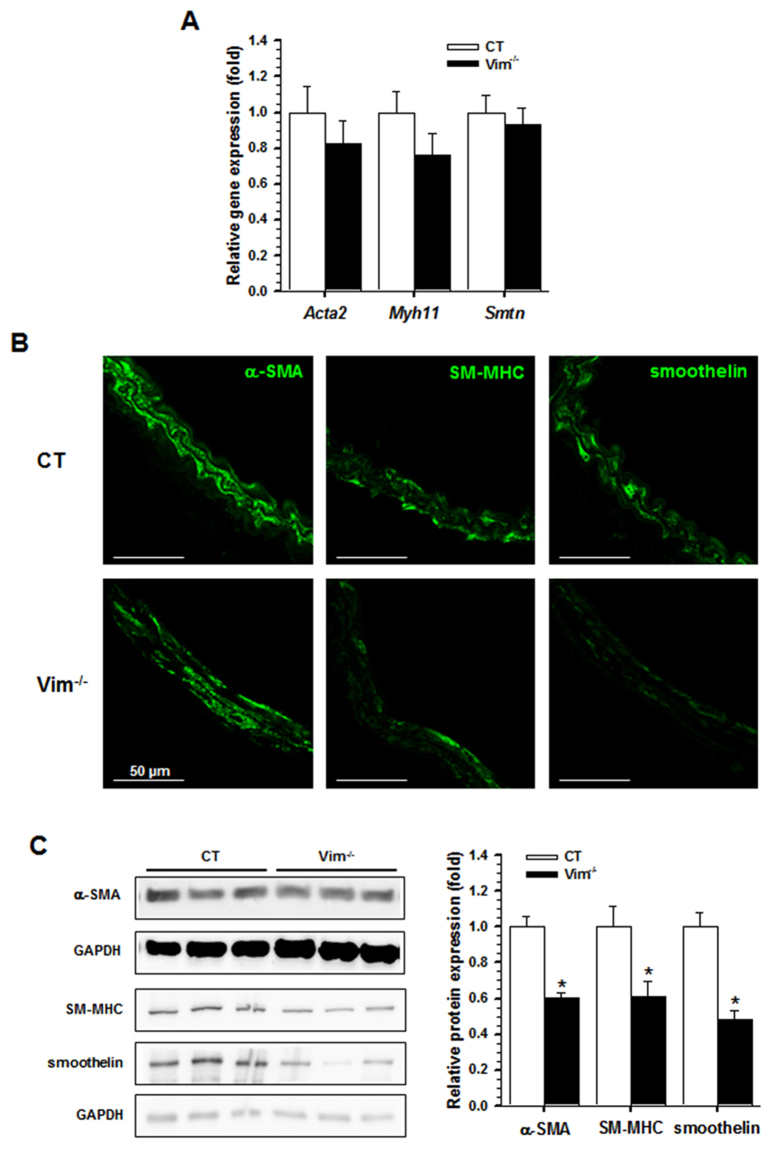
Alteration of specific markers of smooth muscle cells in the carotid artery. (**A**) Relative mRNA levels (Vim^−/−^ vs CT) of genes encoding α-SMA, SM-MHC and smoothelin in the carotid artery by qRT-PCR. (**B**) Immunofluorescence staining shows a decrease in α-SMA, SM-MHC and smoothelin in Vim^−/−^ mice. (**C**) Western blot analysis shows a decrease in α-SMA, SM-MHC and smoothelin of SMCs from Vim^−/−^ mice. Results are expressed as means ± SEM (n ≥ 3 in each group). *P < 0.05 compared with CT mice by the unpaired Student’s t-test. Scale bars = 50 µm in panel B.

We tested whether basement membrane changes were associated with changes in markers of the various stages of vascular SMC differentiation (early, midstage and fully differentiated). Expression of SMC-specific genes coding for α-SMA (*Acta2*), SM-myosin heavy chain (*Myh11*) and smoothelin (*Smtn*) was unchanged in Vim^−/−^ mice compared with CT mice (Fig. 4A). Expression of other SMC-specific markers such as caldesmon and calponin as well as the SMC acto-myosin regulatory proteins Myl9, MYPT1, CPI-17, cofilin and RhoA was unchanged in Vim^−/−^ mice compared with CT mice at both the mRNA and protein levels (Supplementary Fig. S2). However, a marked decrease in α-SMA, SM-myosin heavy chain and smoothelin protein levels was observed by immunofluorescence staining and/or Western blot analysis (Fig. 4B,C). This decreased expression demonstrates that the loss of Vim may have influenced the stability of the protein markers of the SM contractile phenotype, indicating a less differentiated state of vascular SMCs.

### Increased expression of focal adhesion proteins in Vim^−/−^ mice

**Figure 5:**
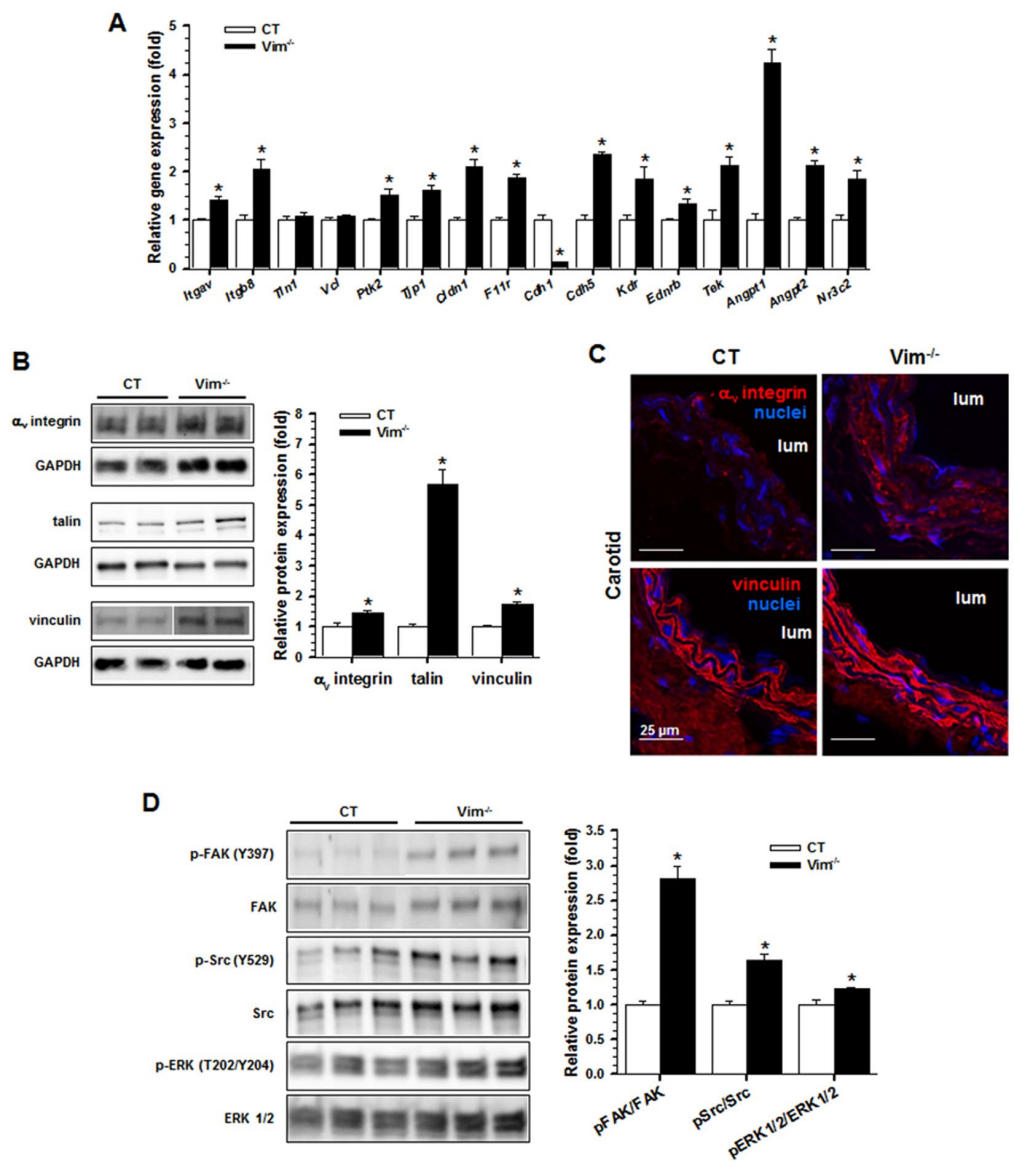
Focal adhesion proteins in the carotid artery. (**A**) Relative mRNA levels (Vim^−/−^ vs CT) of genes encoding components of focal adhesions by qRT-PCR. (**B**) Western blot analysis shows the increased expression of α_v_ integrin subunit, talin and vinculin in Vim^−/−^ mice. Results are expressed as means ± SEM (n ≥ 3 in each group). (**C**) Immunofluorescence staining shows increased α_v_ integrin and vinculin in SMCs from Vim^−/−^ mice. (**D**) Western blot analysis shows an increase in phosphorylated FAK, Src and ERK1/2 in Vim^−/−^ mice. Results are expressed as means ± SEM (n = 7 in each group). *P < 0.05 compared with CT mice by the unpaired Student’s t-test. Scale bars = 25 µm in panel C.

To explore whether modifications in differentiation markers of both ECs and SMCs impact FA activation, we profiled the expression of genes involved in focal adhesions. Expression of genes encoding the integrin subunits α_v_ and β_8_ (*Itgav*, *Itgb8*), components of FAs including focal adhesion kinase (*Ptk2*), ZO-1 (*Tip1*), claudin (*Cldn1*), JAM-1 (*F11r*), VE-cadherin (*Cdh5*), VEGFR2 (*Kdr*), EDNRB (*Ednrb*) and Tie2 (*Tek*) receptors, the cytokines ANG1 (*angpt1*) and ANG2 (*Angpt2*), as well as the mineralocorticoid receptor (*Nr3c2*) was increased in Vim^−/−^ mice compared with CT mice (Fig. 5A). Increases in the α_v_ integrin subunit, talin and vinculin were observed at the protein level (Fig. 5B). Immunofluorescence analysis showed also an increase in vinculin and confirmed the increase in the α_v_ integrin subunit (Fig. 5C).

To analyze whether loss of vimentin also modified the proteins involved in linking integrin adhesion molecules to the actin cytoskeleton, we quantified phosphorylation of FAK, Src, and ERK1/2. We found a higher level of phosphorylation of FAK, Src and ERK1/2 in the Vim^−/−^ mice compared to the CT mice (Fig. 5D). Taken together, these data suggest that Vim knockout promotes increased expression of FA proteins and phosphorylation of target proteins in FA signaling.

### Knockout of vimentin results in decreased endothelial relaxation and in carotid stiffening

**Figure 6:**
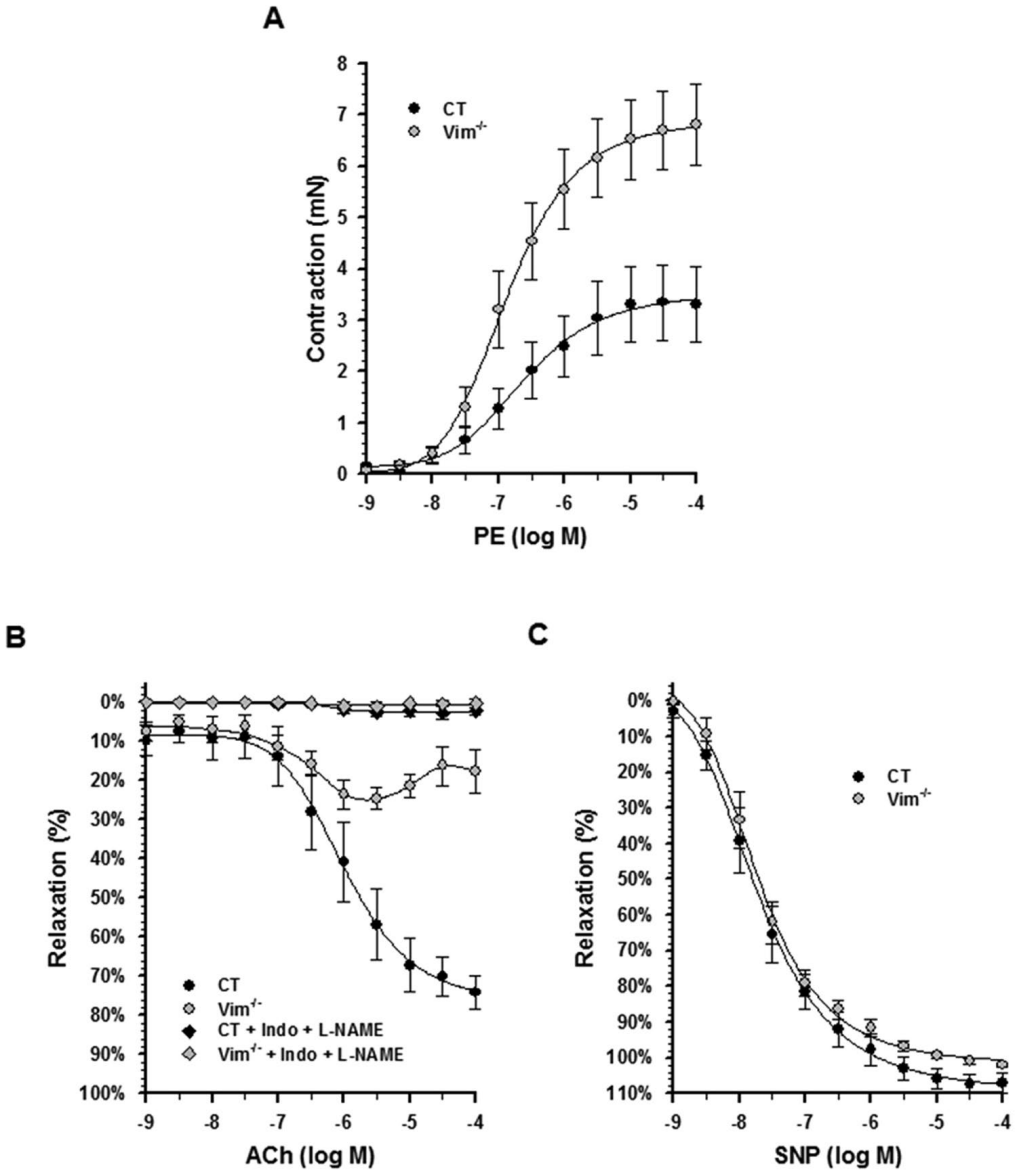
Vascular responses to vasomotor agents in the aorta from Vim^−/−^ and CT mice. (**A**) Contraction induced by different concentrations of phenylephrine (PE) in intact aortic rings from CT (n = 7) and Vim^−/−^ (n = 6) mice. (**B**) Endothelium-dependent relaxation in response to different concentrations of acetylcholine (ACh) in either the absence or presence of an inhibitor of cyclooxygenase 1 and 2, indomethacin (10^−5^ M) and a NO inhibitor, L-NAME (10^−4^ M) in PE-precontracted aortic rings from Vim^−/−^ and CT mice (n = 7 in each group). (**C**) Endothelium-independent relaxation induced by different concentrations of the NO donor, sodium nitroprusside (SNP), in PE-precontracted aortic rings from Vim^−/−^ and CT mice (n = 7 in each group).

Because changes in contractile protein expression and structural alterations of endothelium may act on vasomotor tone in opposite ways, aortic function was explored *in vitro* using wire myography. There was a significant increase in phenylephrine-induced contractile responses of aortic rings from Vim^−/−^ mice compared with rings from CT mice (Fig. 6A), characterized by an increased maximal efficiency (E_max_, 7.3 ± 0.8 versus 3.7 ± 0.9 mN; p < 0.05) with no change in the negative logarithms of the concentration required to produce 50% contraction (pD2, −6.7 ± 0.1 versus −6.4 ± 0.2). The vasodilatory response to acetylcholine (ACh) was decreased (Fig. 6B) in Vim^−/−^ rings compared to CT rings, with a significant decrease in E_max_ (32.7 ± 4.4 versus 77.5 ± 4.6%; p < 0.05) but identical pD2 values (−6.1 ± 0.3 in each group). Indomethacin at 10^−5^ M in addition to Nω-nitro-l-arginine methyl ester (L-NAME) at 10^−4^ M totally abolished ACh-induced relaxation in the 2 groups (E_max_ = 3.2 ± 1.5 versus 1.8 ± 1.2%). The maximal relaxation induced by sodium nitroprusside (SNP) was similar (E_max_ = 107.9 ± 2.7 versus 101.8 ± 0.5%. pD2 = −7.7 ± 0.1 versus −7.6 ± 0.1%) between the 2 groups (Fig. 6C), indicating an endothelium-dependent alteration of relaxation.

To further identify the functional consequences of Vim knockout on hemodynamics *in vivo*, we studied live tissues for biomechanical parameters. Because Synm associates with Vim in arteries, we wished to assess whether knockout of Synm could also affect the hemodynamic and stiffness parameters of Vim^−/−^ mice. Systolic arterial pressure (SAP) in conscious Vim^−/−^ mice was lower than in CT mice. SAP, diastolic arterial pressure (DAP) and mean arterial pressure (MAP) in anesthetized Vim^−/−^ mice were significantly lower than in CT mice (Table 1). SAP, DAP and MAP were lower in anesthetized Vim^−/−^ Synm^−/−^ mice compared with CT mice whereas there were no differences between Synm^−/−^ and CT mice and between Vim^−/−^ and Vim^−/−^ Synm^−/−^ mice (Table 1).

**Table 1:** Blood pressure, mechanical properties and composition of the carotid artery

	CT	Synm^−/−^	Vim^−/−^	Vim^−/−^Synm^−/−^
Number	7	6	6	4
*Conscious mice*				
SAP (mmHg)	109 ± 4	107 ± 4	91 ± 4*	99 ± 4
*Anesthetized mice*				
SAP (mmHg)	113 ± 5	109 ± 5	91 ± 2*	100 ± 4
DAP (mmHg)	79 ± 4	75 ± 5	62 ± 2*	66 ± 4
MAP (mmHg)	91 ± 4	87 ± 5	72 ± 1*	77 ± 4
PP (mmHg)	34 ± 3	34 ± 3	29 ± 3	34 ± 3
HR (beats/min)	438 ± 14	446 ± 18	397 ± 19	401 ± 17
*Parameters within common range*				
MDia_80–88_ (µm)	510 ± 32	531 ± 34	531 ± 20	522 ± 49
MDist_80–88_ (10^−3^ mmHg^−1^)	13.5 ± 1.9	12.4 ± 2.0	6.3 ± 0.3*^§^	6.9 ± 1.2*^§^
MWS_400–800_ (kPa)	250 ± 22	245 ± 18	194 ± 5*^§^	174 ± 12*^§^
*Histology of the CA*				
MCSA (mm^2^ 10^−3^)	25 ± 2	27 ± 1	24 ± 2	31 ± 1*

Values are means ± SEM; CT, control mice; DAP, diastolic arterial pressure; MAP, mean arterial pressure; PP, pulse pressure; MDia, mean diameter; MDist, mean distensibility; MWS, mean wall stress. *P < 0.05 versus CT §P < 0.05 versus Synm^−/−^.

**Figure 7:**
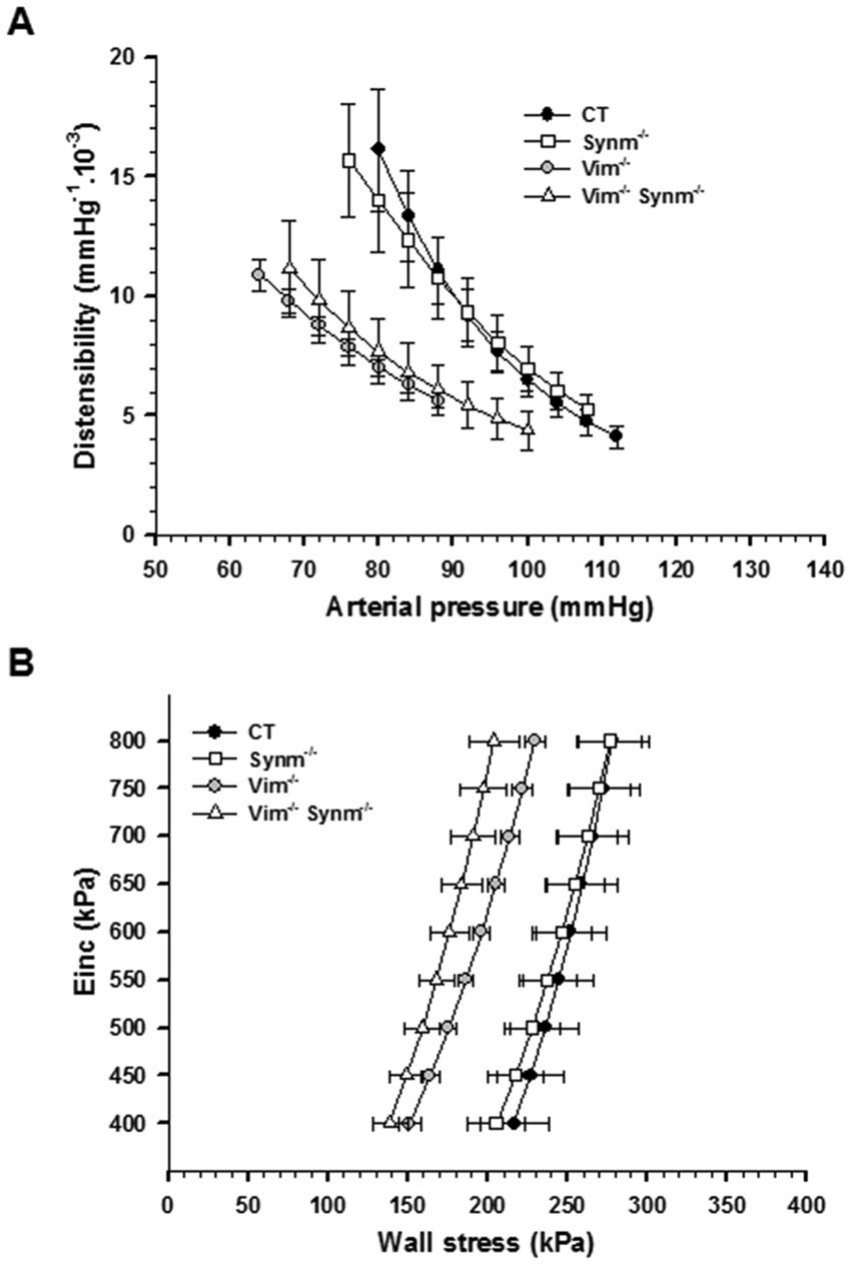
Mechanical properties of the carotid artery from CT and knockout (Synm^−/−^, Vim^−/−^ and Vim^−/−^ Synm^−/−^) mice. Distensibility-arterial pressure curves (**A**) and incremental elastic modulus (Einc)-wall stress curves (**B**) in CT (n = 7), Synm^−/−^ (n = 6), Vim^−/−^ (n = 6) and Vim^−/−^ Synm^−/−^ (n = 4) mice.

Pulse pressure and heart rate were not different between the four groups. The mean arterial diameter (MDia_80_–_88_) and lumen cross-sectional area distensibility (MDist_80–88_) within the 80–88 mmHg common range of arterial pressure were calculated from the Diameter (Dia)/arterial pressure curves (not shown) and Distensibility (Dist)/arterial pressure curves shown in Fig. 7A. MDia_80–88_ was not different between groups. In Vim^−/−^ and Vim^−/−^ Synm^−/−^ mice, the Dist-arterial pressure curves were significantly shifted downwards from those of Synm^−/−^ and CT mice. The incremental elastic modulus (Einc) / circumferential wall stress (WS) curves are shown in Fig. 7B. The Einc-WS curves of Vim^−/−^ and Vim^−/−^ Synm^−/−^ mice were shifted significantly leftwards compared with the 2 other groups. MDist_80–88_ and the mean WS within the 400–800 kPa range of Einc (MWS_400–800_) were decreased in Vim^−/−^ and Vim^−/−^ Synm^−/−^ mice compared to Synm^−/−^ and CT mice. Media cross sectional area (MCSA) of the carotid artery was increased in Vim^−/−^ Synm^−/−^ mice as compared with CT mice.

Thus, knockout of the Vim gene alone induced an increase in arterial stiffness with both structural and functional alterations of vascular tone.

## Discussion

This study demonstrates that Vim^−/−^ mice exhibited increased expression of components of the subendothelial basement membrane and a decreased expression of mature SMC differentiation markers. Our study also highlights increased expression of key FA-associated proteins. The main functional consequence is an increase in vascular tone and an alteration of endothelium-dependent relaxation, leading to an increase in carotid stiffness in adult Vim^−/−^ mice, in the absence of hypertension and changes in the elastin/collagen ratio. Our data show that Synm alone has no influence on carotid stiffness. Overall, our study demonstrates that IFs located both in ECs and VSMCs act in concert to regulate cell structure and mechano-transduction pathways.

In rats and mice, most studies that have focused on cellular and molecular determinants of arterial stiffness have looked at local elastic artery distensibility and Einc to analyze directly the structure/function relationship. This is particularly useful to study the effects of genetic manipulations of molecules such as integrins or cytoskeletal proteins involved in endothelial or SMCs. Trachet *et al*. have shown that in mice, determination of aortic stiffness using pulse wave velocity should be avoided and replaced by local stiffness methods^37^. Arterial distensibility was compared at equivalent blood pressure levels within the cardiac cycle. Regarding the intrinsic mechanical properties, Einc changes are independent of geometry and blood pressure changes^38^.

From a functional point of view, blood pressure levels and arterial parameters of Vim^−/−^ Synm^−/−^ mice were not different from those of Vim^−/−^ mice. This finding is largely explained by the fact that Synm requires an appropriate co-polymerization partner, such as Vim in smooth muscle, Des in skeletal and cardiac muscle or keratin IF to form filament networks^39,40^. The absence of Synm in the carotid of Vim^−/−^ mice confirmed that Synm associates with Vim in vascular cells^10,40^. The slight medial hypertrophy observed in Vim^−/−^ Synm^−/−^ mice has been observed previously during skeletal muscle hypertrophy through regulation of protein kinase A and Akt signaling in response to mechanical load^41^. The slight reductions in SAP in conscious mice are likely not related to changes in vasomotor tone and arterial stiffness since our data rather predict an increase in blood pressure. Einc characterizing the stiffness of the wall material is independent of the size of the artery, its thickness and blood pressure changes. In Vim^+/+^ mice, knockout of Synm had no effect on mechanical properties, indicating that the role of Synm is negligible compared to the role of Vim.

The contribution of Vim to cell mechanical properties is complex and depends on substrate stiffness. It was reported in living cells in culture that Vim-deficient lymphocytes, fibroblasts and glial cells are more deformable indicating a lower cellular stiffness^35,42–44^. However, using atomic force microscopy, it has since been revealed that Vim protects against compressive stress and preserves viscoelastic properties of mouse embryonic fibroblasts under high repetitive strain^36^. In support of a role for IF in compression, disruption of IFs by acrylamide decreased strain-dependent compressibility in chondrocytes^45^. The vascular wall is presumed to be incompressible and this assumption is integrated in the determination of the elastic modulus/wall stress curves^38^. Under physiological conditions, ECs and vascular SMCs are constantly submitted *in vivo* to pulsatile mechanical forces. Therefore, we can anticipate that pulsatility, even at low mean arterial pressure and unchanged collagen amounts, contributes to the increased stiffness in Vim^−/−^ mice.

The current concept is that the elastin/collagen ratio and cell-matrix interactions are the main factors influencing stiffness of large arteries^22,23,46^. Our finding of a marked increase in fibronectin, laminin, collagen IV and perlecan in Vim^−/−^ mice, predominantly in the basement membrane underlying the endothelium, is in accord with the role of medial fibronectin accumulation in arterial stiffness reported both in SHR and aldosterone-salt models of hypertension^23,38^. The higher level of global stiffness of the basement membrane compared to adjacent cells^47–50^ argues for a physiological relevance of the basement membrane in arterial stiffness. The asymmetric organization of basement membrane proteins provides site-specific differences in the biomechanical properties of cells^49,51^. In the eye, it has been reported that the epithelial site is stiffer than the stromal site^49,51^. In large arteries, there are limitations to analyze these site-specific properties in vascular stiffness changes. However, the relevant functional marker of basement membrane stiffness is its density, which is increased in Vim^−/−^ mice as assessed by electron microscopy. Laminin, one of the major functional components of basement membranes, is considered to provide the main cell binding activity of basement membranes, and the collagen IV network is considered as the main stabilizing structure. The increase in thickness as observed by immunofluorescence staining may be attributed to heparan sulfate proteoglycan accumulation since perlecan has been proposed to bind growth factors and to regulate the hydration status of basement membranes. When compared with previous data reported in Des^−/−^ mice^33^, the 53% decrease in distensibility in Vim^−/−^ mice is 1.8-fold more than the 29% decrease in Des^−/−^ mice, indicating that vascular stiffness is higher in Vim^−/−^ mice than in Des^−/−^ mice. It should be emphasized that Des is present only in SMCs while Vim is present in both SMCs and ECs. The observation that in Des^−/−^ mice, only collagen IV expression was increased (supplementary Fig. S1) indicates a crucial role of basement membrane laminin, fibronectin and perlecan in the stiffness of Vim^−/−^ mice. The increase in these proteins may reflect endothelial activation, a hypothesis supported by the increase in specific markers such as VWF and VE-cadherin. In addition, location of part of VWF below perlecan staining suggests strongly an important structural reorganization leading to stiffening of the subendothelium. It has been reported that subendothelial matrix stiffening is necessary and sufficient to promote endothelial activation *in vitro*^52^. However, the increased vasoconstriction and decreased endothelium-dependent relaxation supports the hypothesis that vasomotor tone contributes to arterial stiffening in Vim^−/−^ mice. This is also in agreement with previous studies showing that mechanical de-endothelialization in rats or knockout of Des increases the viscous behavior of the arterial wall^33,53^. Whether these biomechanical changes are chronically associated with endothelial basement membrane remodeling remains to be investigated.

The contribution of vascular SMCs to arterial stiffness has been assessed by the expression of specific markers of mature smooth muscle cells and cell-matrix interactions. In Vim^−/−^ mice, the decreased expression of SM-actin, SM-MHC and smoothelin at the protein level, together with a decrease in finger-like projections, indicates a SMC phenotypic change towards a less differentiated state. Decreased SM-actin, SM-MHC and smoothelin at the protein level, but not at the mRNA level, in Vim^−/−^ mice suggests that vimentin could influence the stability of these proteins. It has been suggested that plectin sidearms on IFs link them to the microtubules, actomyosin in stress fibres and membrane components. Cells lacking vimentin present an altered interaction of plectin-microtubule and plectin-myosin^54^. In Des^−/−^ mice, deficient SMC-matrix interactions related to SMC dedifferentiation resulted in increased arterial stiffness. The increased arterial stiffness in Vim^−/−^ mice, also exhibiting a reduction in finger-like projections, is again consistent with a role of cell-matrix interactions in arterial stiffening.

The relationship between a reduction in actomyosin content and contractility appears to be very complex. Indeed, excitation/contraction coupling is not only dependent on the degree of differentiation but also on the general molecular environment of SMCs. Recently it has been reported in cancer cells and fibroblasts that vimentin depletion induced phosphorylation of the microtubule-associated GEF-H1, and thereby increased RhoA activity, myosin light chain phosphorylation, actin stress fiber assembly and cell contractility^55^. In the present study, the expression of genes coding for RhoA, cofilin, calponin 1, caldesmon, myosin regulatory light polypeptide 9 and the regulators of myosin light phosphatase MYPT1 and CPI-17 remained unchanged at both the mRNA and protein levels. The thicker endothelial cell basement membrane and the increase in subendothelial matrix synthesis are unlikely to enable bioactive compounds abnormally released from non-vascular tissues due to vimentin knockout to enter the media by radial conductance from the blood. Our data are thus more in favor of intravascular mechanisms of Vim knockout-mediated arterial stiffening even though such compounds could, in theory, alter endothelial function and gene expression in SMCs.

Vim plays an important role in the turnover of FAs and in the process of formation and release of integrin endocytic vesicles. Vim, highly expressed in ECs of adult mice, is a component of junctional complexes that couple VE-cadherin to both actin and the IF cytoskeleton^56^ and to FAs^16–19^ where it binds to integrins via VAMs^20,21^. In vascular SMCs, activation and the dynamic activity of FA are likely involved in aortic stiffness^57^. Changes in mRNA in Vim^−/−^ mice support the hypothesis of an increase in FA turnover mainly in ECs. Indeed, the increased expression of genes coding for components of the angiopoietin-2/Tie2/α_v_β_3_ integrin axis in Vim^−/−^ mice suggests that the loss of Vim stimulates FA turnover in ECs via internalization and degradation of α_v_β_3_^58^. There is no specific role in FA for E-cadherin, whose mRNA level is decreased markedly, except in cancer-induced cell proliferation where its expression is associated with Vim^59^. The increases in mRNA of genes encoding VE-cadherin and VEGFR2 may rather limit EC permeability via enhanced formation of VE-cadherin/VEGFR2 complexes in adherent junctions^60^. Increased mRNA for claudin, ZO-1 and JAM-1 may participate also in the regulation of the EC barrier function. The increase in *Ednrb* mRNA is consistent with a role of the encoded receptor in FAK and paxillin phosphorylation, thus favoring FA formation^61^.

In vascular SMCs, our results show clearly an increase in the three main protein entities of FA, α_v_β_3_ integrin, talin and vinculin. These observations are in line with the reported role of Vim in reducing FA size but not FA half-life in metastatic fibroblast cells expressing high levels of endogenous Vim^34^. An enlargement of FA has also been shown in cultured Vim-deficient fibroblasts independently of tension conditions^5^. In Vim^−/−^ mice, the increase in phosphorylation of the major mechanosensory molecule, FAK, and its downstream targets, Src and ERK1/2, resulting from integrin engagement is consistent with an increase in FA assembly and disassembly rates. Assuming that the number of SMCs is higher than the number of ECs in the entire vascular wall, these data suggest that activation of FAs in the media, is the major contributor to the increased arterial stiffness in Vim^−/−^ mice. One limitation of our study is that we did not measure SMC stiffness that could be increased regarding the changes of these FA proteins. However, more generally, the exact contribution of cell stiffness to that of the global arterial wall remains unclear and requires further *in vivo* and *in vitro* experiments. Taken together, our results suggest that in carotid arteries under physiological pressure and flow, Vim, but not Synm levels, control subendothelial matrix stiffening, vasoreactivity, vascular SMC differentiation and FA turnover. Hence, our study identifies a new mechanistic role of Vim IF in regulating cell-matrix interactions in arterial stiffness.

## Material and Methods

### Animals

Mice, knocked out for Vim (Vim^−/−^), synemin (Synm^−/−^) and desmin (Des^−/−^) on a C57BL/6 N background, were obtained and genotyped by PCR as described previously^33,41,62^. All mice were housed in a temperature- and humidity-controlled facility with a 12 h light/day cycle. In this study, six-month old Vim^−/−^, Synm^−/−^, and Vim^−/−^ Synm^−/−^ mice were used for *in vivo* arterial mechanical property measurements. Wild-type and heterozygous mice were used as controls (CT). Des^−/−^ mice were used to analyze Vim and Synm colocalization in carotid arteries. For molecular and cellular analysis, age-matched (Vim^−/−^ and CT) mice were used. All animal studies were approved by the Organ for Prevention and Wellbeing of Animals of Institut National de la Santé et de la Recherche Médicale (INSERM) and the Comité d’Ethique Lorrain en Matière d’Experimentation Animale (CELMEA) and conducted according to French and European laws, directives, and regulations on animal care.

### *In vivo* arterial mechanical parameters

Systolic arterial pressure (SAP) and heart rate (HR) were measured in conscious animals using a tail-cuff sphygmomanometer (Hatteras Instruments, Inc., NC, USA). With an ultrasonic echotracking device (NIUS-01, Asulab SA, Neuchâtel, Switzerland), we recorded arterial diameter (Dia, left carotid artery) and arterial pressure (right carotid artery) simultaneously in isoflurane-anesthetized mice as described previously^30^. The relation between arterial pressure (AP) and lumen cross-sectional area (LCSA) was fitted by an arctangent function. LCSA distensibility (Dist), a derivative of this function, was used to assess global elastic behaviour of the artery. Circumferential wall stress (WS) and incremental elastic modulus (Einc), which characterizes the intrinsic mechanical properties of the wall material, were calculated with the above-mentioned parameters and the media cross sectional area (MCSA).

### Vascular reactivity analysis

Vascular contractile and relaxing responses were assessed in isolated thoracic aortas from Vim^−/−^ and CT mice as described previously^30^. Briefly, aortas were excised, cut into 2.5 mm segments and mounted in a wire myograph (DMT, Aarhus, DK) in an 8 mL organ bath containing oxygenated physiological salt solution (pH 7.4) maintained at 37 °C. Rings were allowed to equilibrate for 30 min at a resting tension of 1 g, changing the bath medium every 10 minutes. All preparations were contracted maximally with an isotonic KCl solution (80 mM) and then with a submaximal concentration of phenylephrine (PE, 10^−5^ M) to assess contractile capacity of each ring. The α-adrenergic pathway was assessed via the contraction induced by PE (10^−9^ to 10^−5^ M). To evaluate endothelium-dependent relaxation, the dose response to acetylcholine (ACh, 10^−9^ to 10^−4^ M) was determined in rings precontracted by 10^−5^ M PE, in the presence or absence of 10^−5^ M indomethacin, an inhibitor of cyclooxygenase (COX) 1 and 2 enzymes plus 10^−4^ M Nω-nitro-l-arginine methyl ester (L-NAME), a nitric oxide (NO) inhibitor. Vascular SMC sensitivity to the NO pathway was also assessed by the dose-response to the NO donor sodium nitroprusside (SNP, 10^−9^ to 10^−4^ M) in rings precontracted with PE (10^−5^ M). The maximal response (E_max_) and the concentration of agonist inducing 50% of the maximal response (EC_50_) were extrapolated from the individual concentration-effect curves. These latter values were transformed into pD2 values, i.e. negative logarithms of EC_50_ values.

### Histological Procedure**s**

Histological studies were performed on carotid arteries (CA) fixed with 10% buffered formalin under pressure *in vivo*. For morphological analysis, all arterial samples were embedded in paraffin and 5 μm sections were stained with Sirius red for collagen and Weigert’s resorcin-fuchsin for elastic fibres. The density was calculated by drawing boxes of equal size on each cross-section of carotid artery. The intensity of staining was averaged over 5 to 7 boxes/sample for Weigert’s resorcin-fuchsin staining and 10 boxes/sample for Sirius red staining, for each mouse artery fixed under pressure. Composition of the arterial wall and the MCSA were determined by computer-directed image analysis.

### Immunohistochemistry

Seven-micrometer transverse frozen sections were prepared from carotid arteries and aortas of mice as described previously^30^. The sections were incubated with primary antibodies (Supplementary Table 1) against fibronectin, E-cadherin, SM-MHC, laminin, Perlecan, α_v_ integrin subunit, Collagen IV. α-SM-actin, smoothelin, Vim, Synm, Des, CD31, von Willebrand factor (VWF) and VE-cadherin. After washing in PBS, sections were incubated for 1 hour with secondary antibodies (Life Technologies, Saint Aubin, France). Nuclei were counterstained with DAPI or DRAQ5. After washing in PBS, slides were mounted using fluoromount aqueous medium (Sigma). Images were captured using a confocal laser-scanning microscope (Zeiss SP5, France) and basement membrane thickness was determined from 3 different mice.

### Quantitative real-time PCR

Total RNAs were extracted from carotid arteries using TRI Reagent (Sigma, St. Louis, MO, USA) and were reverse transcribed with the high capacity cDNA reverse transcription kit (Applied Biosystems) and random hexamers to generate cDNAs. PCR analysis was then performed with SYBR green PCR technology (Roche). The Primer3 program (frodo.wi.mit.edu/primer3/) was used to select primers (Supplementary Table 2) and the housekeeping gene, GAPDH, was used to normalize expression levels.

### Western blot analysis

Immunoblotting was carried out as described previously^41^ using carotid arteries or aortas from Vim^−/−^ and control mice. Vessels were snap-frozen in liquid nitrogen immediately after dissection. Frozen vessels were placed into an ice-cold homogenization buffer containing: 50 mM Tris (pH 7.6), 250 mM NaCl, 3 mM EDTA, 3 mM EGTA, 0.5% NP40, 2 mM dithiothreitol, 10 mM sodium orthovanadate, 10 mM NaF, 10 mM glycerophosphate and 2% of protease inhibitor cocktail (Sigma-Aldrich, Saint-Quentin Fallavier, France). Samples were homogenized using an ultraturrax, incubated 30 min on ice and then centrifuged at 12,000 *g* for 20 min at 4 °C. Protein concentrations were measured using the Bradford method with bovine serum albumin as standard. Equal amounts of protein extracts (15 µg) were separated by SDS-PAGE before electrophoretic transfer onto a nitrocellulose membrane (Amersham Hybond-ECL, GE Healthcare, Velizy-Villacoublay, France).

### Electron microscopy

The carotid artery was isolated from mice and fixed in a Carson solution of pH 7.2 (3.5% formaldehyde, 110 mM Na_2_HPO_4_). All samples were processed as described previously^30^ and embedded in Epon. Ultrathin sections were observed with a Zeiss omega TEM microscope. Quantitative analysis on TEM images was performed by using ImageJ 1.46r, developed at the National Institutes of Health, USA, and freely available at https://imagej.nih.gov/ij/. To quantify the mean intensity of the subendothelial basement membrane, arbitrarily chosen fragments underneath 6–16 cells from triplicate mice where measured along a straight line; the mean gray value obtained was subtracted from the background, corresponding to the artery lumen, and normalized as a fold induction over the control mean value (i.e. the gray value of a wt tissue) acquired with the same settings in the same microscopy session.

### Statistical analysis

All values are expressed as means ± SEM. Differences between groups were assessed with the unpaired Student’s t-test. Dist and Einc were log transformed to generate linear relations^63^. The quality of the transformation was checked by calculating the R² of the linear regression obtained with the new parameters for each individual. After this transformation, we calculated the mean slopes of the curves. If the slopes were not significantly different, we compared the curves by calculating the median values of the common range of either AP for Dia, Dist or Einc for WS. AP and arterial mechanical parameters were analysed using Kruskal-Wallis multiple-comparison tests. Differences were considered significant at values of p < 0.05.

### Data availability

All data generated or analysed during this study are included in this published article and its Supplementary Information files.

## Electronic supplementary material

Supplementary data
