# Publisher Correction: Vimentin knockout results in increased expression of sub-endothelial basement membrane components and carotid stiffness in mice

**DOI:** 10.1038/s41598-018-22187-y

**Published:** 2018-03-06

**Authors:** Benoit Langlois, Ekaterina Belozertseva, Ara Parlakian, Mustapha Bourhim, Jacqueline Gao-Li, Jocelyne Blanc, Lei Tian, Dario Coletti, Carlos Labat, Zhor Ramdame-Cherif, Pascal Challande, Véronique Regnault, Patrick Lacolley, Zhenlin Li

**Affiliations:** 1 Inserm, UMR_S 1116, Université de Lorraine, Nancy, France; 2 Sorbonne Universités, UPMC Univ Paris 06, INSERM ERL U1164, Institut Biologie Paris-Seine, Paris, CNRS UMR 8256 France; 3 Sorbonne Universités, UPMC Univ Paris 06, Institut Jean Le Rond d’Alembert, Paris, CNRS UMR 7190 France

Correction to: *Scientific Reports* 10.1038/s41598-017-12024-z, published online 14 September 2017

The original version of this Article contained typographical errors in the spelling of the authors Zhenlin Li and Patrick Lacolley, which were incorrectly given as Li Zhenlin and Patrick lacolley respectively. These errors have now been corrected in the PDF and HTML versions of the Article.

