# Supplementary material for: Vimentin knockout results in increased expression of sub-endothelial basement membrane components and carotid stiffness in mice
Source: Sci Rep. 2017 Sep 14;7:11628. doi: 10.1038/s41598-017-12024-z (PMC5599644; doi:10.1038/s41598-017-12024-z)

Supplementary materials

Vimentin knock-out results in increased expression of sub-endothelial basement membrane components and carotid stiffness in mice

Benoit LANGLOIS[1¶], Ekaterina BELOZERTSEVA[1¶], Ara PARLAKIAN[2], Mustapha BOURHIM[1], Jacqueline GAO-LI[2], Jocelyne BLANC[2], Lei TIAN[2], Dario COLETTI[2], Carlos LABAT[1], Zhor RAMDAME-CHERIF[1], Pascal CHALLANDE[3], Véronique REGNAULT[1], Patrick LACOLLEY[1], Zhenlin LI[2*]

[1] Inserm, UMR_S 1116, Université de Lorraine, Nancy, France.
[2] Sorbonne Universités, UPMC Univ Paris 06, CNRS, UMR 8256, INSERM ERL U1164, Institut Biologie Paris-Seine, Paris, France
[3] Sorbonne Universités, UPMC Univ Paris 06, CNRS, UMR 7190, Institut Jean Le Rond d'Alembert, Paris, France

[¶] Equal contribution.

Supplementary Table S1. Antibodies used for immunoblotting and immunofluoresence

Antibody	Source	Dilution	Society
**Western Blot**			
Vim	Guinea pig	1/1000	Progen
Fibronectin	Rabbit	1/500	Sigma-Aldrich
Laminin	Rabbit	1/2000	Sigma-Aldrich
SM-MHC	Rabbit	1/1000	Abcam
Smoothelin	Rabbit	1/250	Santa Cruz
α_v integrin	Rabbit	1/1000	Abcam
talin	Mouse	1/1000	Sigma-Aldrich
vinculin	Mouse	1/1000	Sigma-Aldrich
pFAK (Tyr^{397})	Rabbit	1/1000	Cell Signaling
FAK	Rabbit	1/1000	Cell Signaling
pSrc(Tyr^{529})	Rabbit	1/1000	Biosource
Src	Rabbit	1/1000	Cell Signaling
pERK1/2(Thr^{202}/Tyr^{204})	Rabbit	1/1000	Cell Signaling
ERK1/2	Rabbit	1/1000	Cell Signaling
Cofilin	Rabbit	1/1000	Cell Signaling
RhoA	Rabbit	1/1000	Cell Signaling
Myl9	Rabbit	1/1000	Cell Signaling
CPI-17	Rabbit	1/1000	Abcam
Calponin	Rabbit	1/1000	Milipore
MYPT1	Rabbit	1/1000	Cell Signaling
Caldesmon	Rabbit	1/250	Santa Cruz
GAPDH	Mouse	1/3000	Sigma-Aldrich
**Immunofluoresence**			
Fibronectin	Rabbit	1/100	Abcam
Vinculin	Mouse	1/100	Sigma-Aldrich
SM-MHC	Rabbit	1/300	Cell Signaling
Laminin	Rabbit	1/200	Sigma-Aldrich
α_v integrin	Rabbit	1/300	Abcam
Perlecan	Rat	1/200	Millipore
Collagen IV	Mouse	1/100	Dako
α-SM actin	mouse	1/100	Sigma-Aldrich
Smoothelin	Rabbit	1/100	Santa Cruz
Vimentin	Guinea pig	1/100	Progen
Synemin	Rabbit	1/500	Labo-made
CD31	Rat	1/100	BD Bioscience
VWF	Rabbit	1/300	Dako
VE-cadherin	Rabbit	1/100	BD Bioscience

Supplementary Table S2. Primer sequences

Gene family	Gene	Sense primer (5'-3')	Anti-sense primer (5'-3')
Extracellular matrix	*Col1a1*	actggtacatcagcccgaac	tactcgaacgggaatccatc
proteins	*Col3a1*	accaaaaggtgatgctggac	gacctcgtgctccagttagc
	Col8a1	gaatgcctggaatcaaagga	ggattcccataggtccgatt
	Eln	gacttctgggaacgtttgga	agctttataagccgcagcag
	Fn1	aaggttcgggaagaggttgt	ccgtgtaagggtcaaagcat
	Lama2	ccaagaaggaggctgcatag	ccaggtgttgggaagacact
	Lama4	gcctcctgcctgatgtagag	gacaagatgggttgcttggt
	Lamb1	gttcgagggaactgcttctg	gttcaggcctttggtgttgt
SMC markers	*Acta2*	tgtgctggactctggagatg	gaaggaatagccacgctcag
	Myh11	ggcttcatttgttccttcca	cgagcgtccatttcttcttc
	Smtn	tctcaacagcgagaagctga	tggtcaactcctcgacatca
	Cald1	ccaagccttctgacttgagg	tgggttctttctccgattg
SMC regulatory	*Cfl2*	gccaagtgccatttgatctt	gggaccagtgaaaagggaat
Proteins	*Cnn1*	ggccaagacaaaaggaaaca	ccatctgcagtccaatgatg
	Ppp1r12a	ccgccaaaggctatacagaa	tccactaaaatccgacatgct
	Ppp1r14a	gggacttctggaggcttgt	ctcaggctggggtcatctg
	Myl9	accgcaacagcgccgaggac	caaacatggcgaagacattg
	Rhoa	tccgtcggttttctctccatag	cttctcagatgcaaggctca
Focal adhesion	*Itgav*	gggacagggagaaaggagtc	gattccacagcccaaagtgt
proteins	*Itgb8*	gaggttttgatgccatgctt	tgccaatttgctgtcaagag
	Tln1	gagaatgagacggtggtggt	tccgaagcatctcttcctgt
	Vcl	aggccttcttcctggatgtt	aagaaatagggggagcctga
	Ptk2	gcgatcctattgggagatga	ttttggccttgacagaatcc
	Tjp1	agcaagccttctgcacatct	cagcatcagtttcgggtttt
	Cldn1	tggaagatgatgaggtgcag	ccactaatgtcgccagacct
	F11r	tatgatcctgggctctttgg	gggagaggagaagccagagt
	Cdh1	agccattgccaagtacatcc	aaagaccggctgggtaaact
	Cdh5	ccatcttcctctgcatcctc	caactgctcgtgaatctcca
	Kdr	agagttggtggagcatttgg	taggcagggagagtccagaa
	Ednrb	caggaagaagagcggtatgc	gaggaccaggcagaagactg
	Tek	gatcttacacggtgccgatt	ttagattggaagggccacag
	Angpt1	gatcttacacggtgccgatt	ttagattggaagggccacag
	Angpt2	tccaagagctcggttgctat	agttggggaaggtcagtgtg
	Nr3c2	ctagcacagtggggtccatt	aacgtcgtgagcacctttct
Reference gene	*Gapdh*	aactttggcattgtggaagg	acacattgggggtaggaaca

Supplementary figure S1.

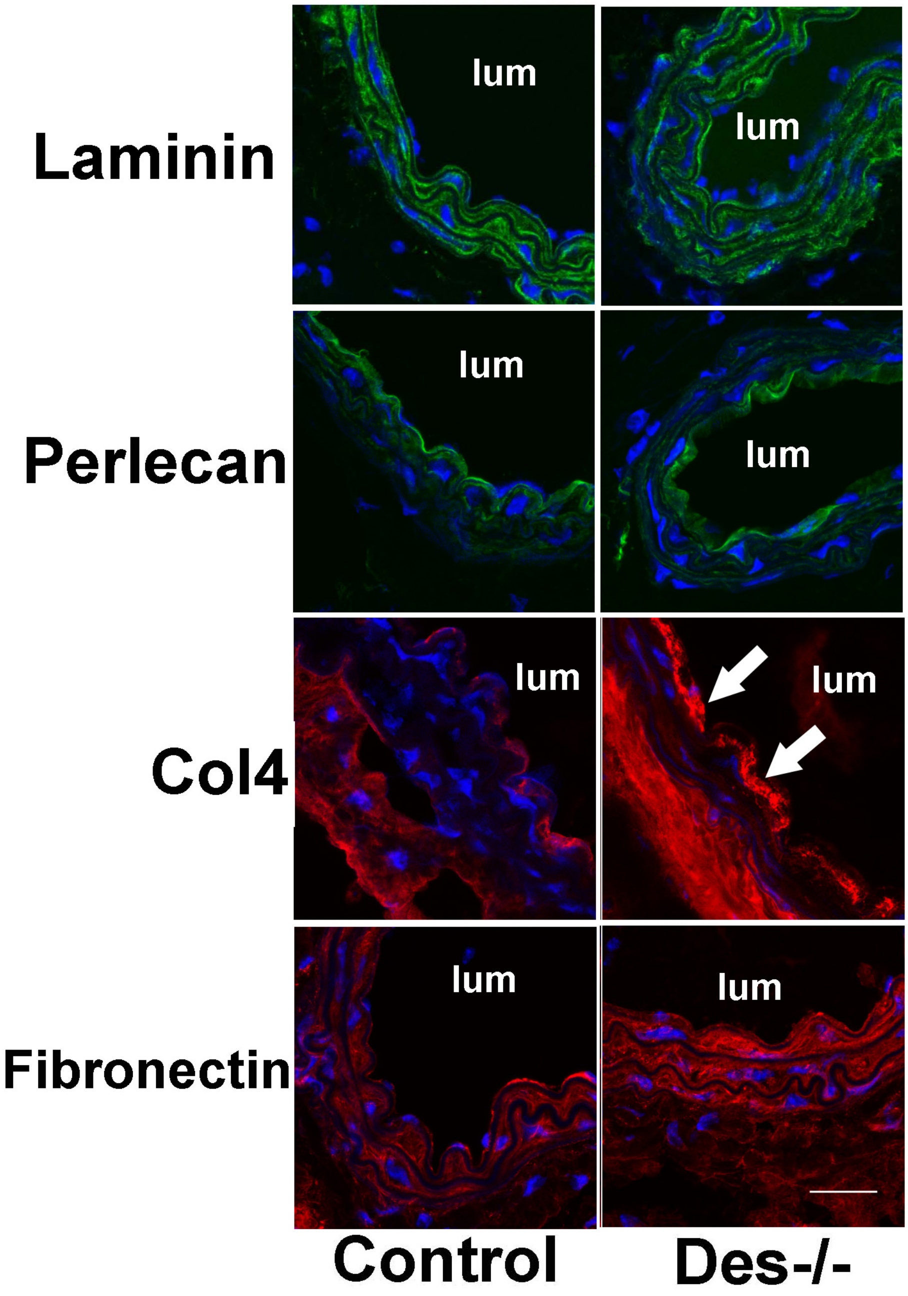

Supplementary figure S1. Confocal immunofluorescence images show the increased expression of only collagen IV (red) indicated by arrows, but not laminin (green), perlecan (green) and fibronectin (red) of basement membrane components in $Des^{-/-}$ mice compared to control mice. Nuclei are stained in blue. Lum: lumen. Scale bars = 25 μm.

Supplementary figure S2.

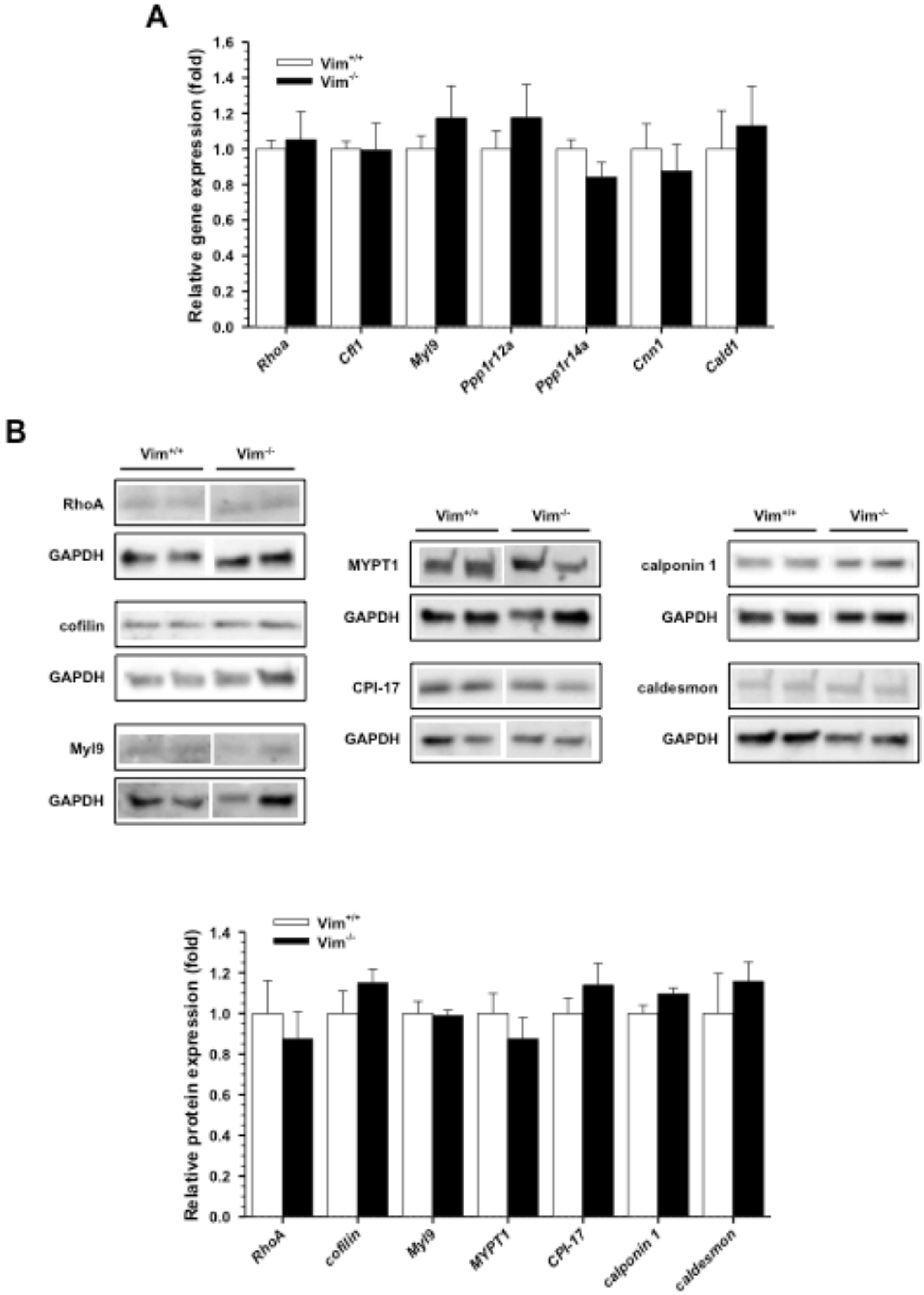

Supplementary figure S2. (A) Relative mRNA levels (Vim$^{-/-}$ vs Vim$^{+/+}$) of genes encoding RhoA, Cfl1 (Cofilin), Myl9, Ppp1r12a (MYPT1), Ppp1r14a (CPI-17), Cnn1 (calponin) and Cadl1 (caldesmon) in the carotid artery by qRT-PCR. (B) Western blot analysis of RhoA, Cofilin , Myl9, MYPT1, CPI-17, calponin and caldesmon in the carotid artery of Vim$^{-/-}$ and Vim$^{+/+}$ mice. Results are expressed as means ± SEM ($n \geq 6$ in each group). No difference of expression of these genes between the Vim$^{-/-}$ and Vim$^{+/+}$ mice at both the mRNA and protein levels.

Western blot used in the figures

Figure 1C

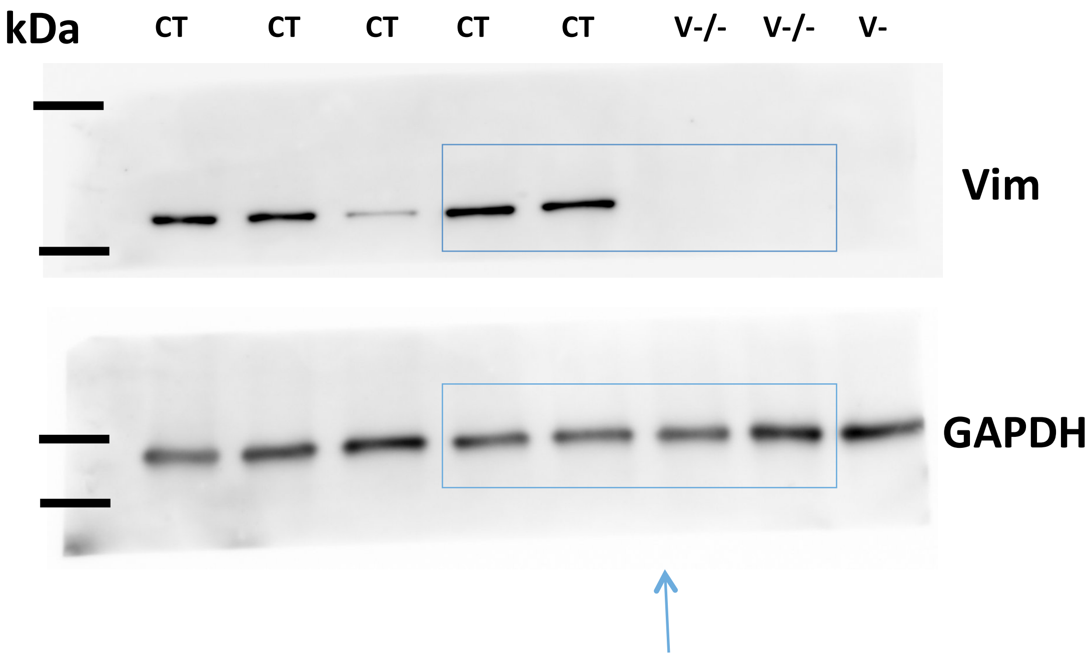

Figure 2D

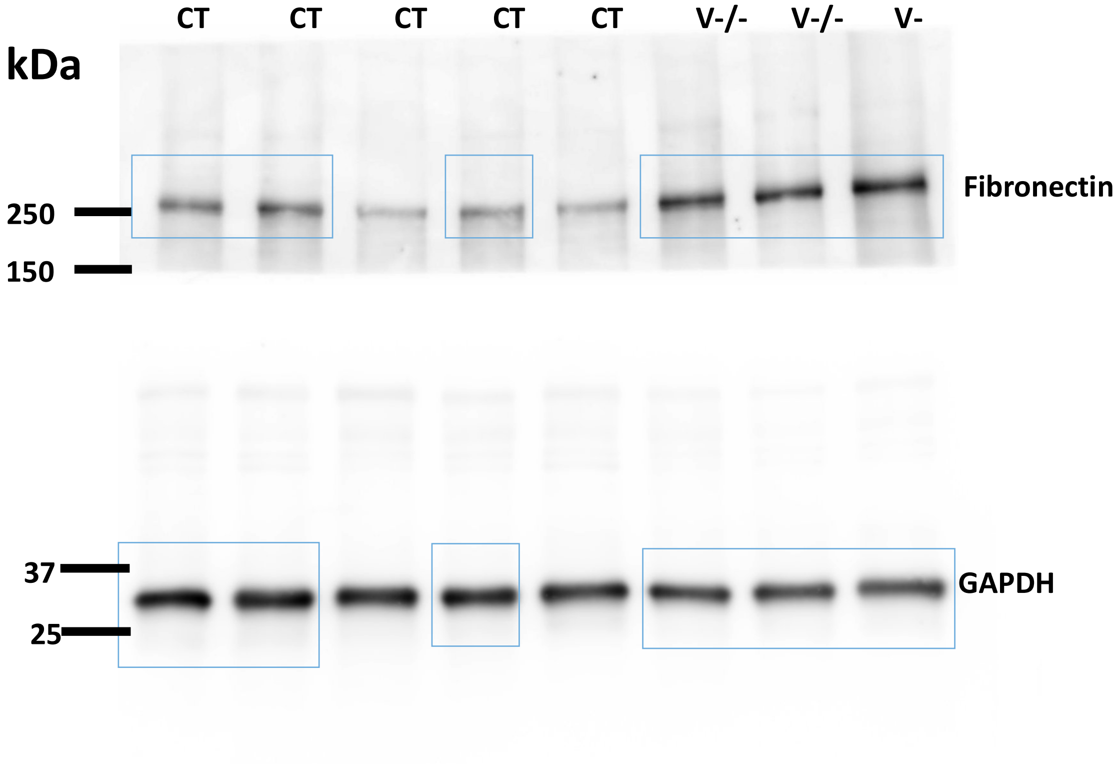

Figure 2D

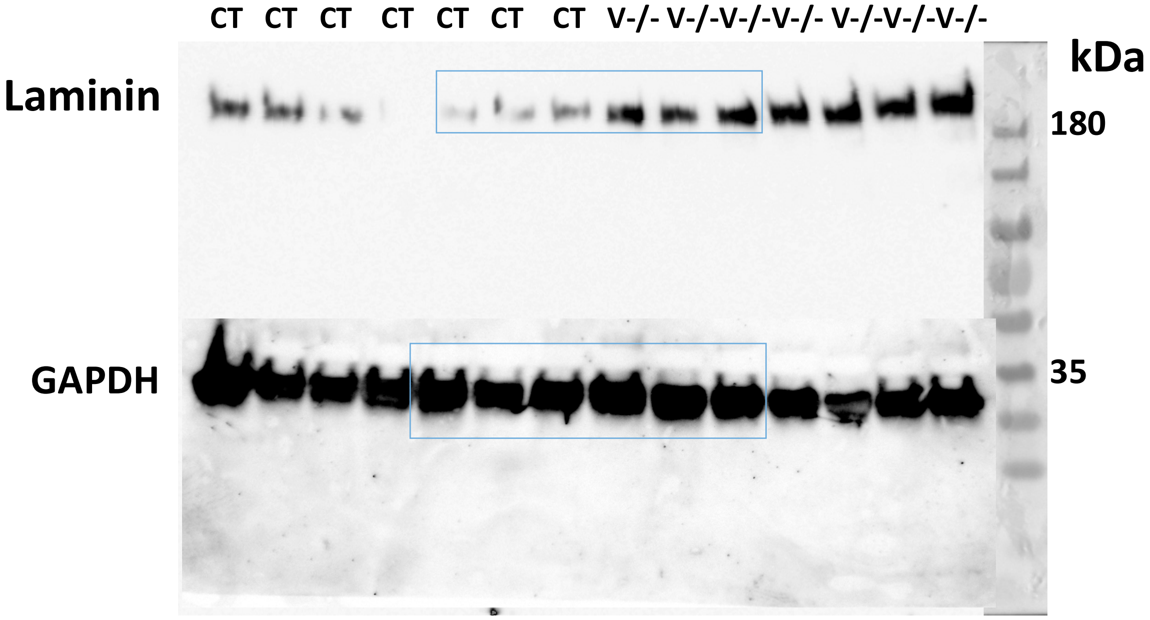

Figure 4C

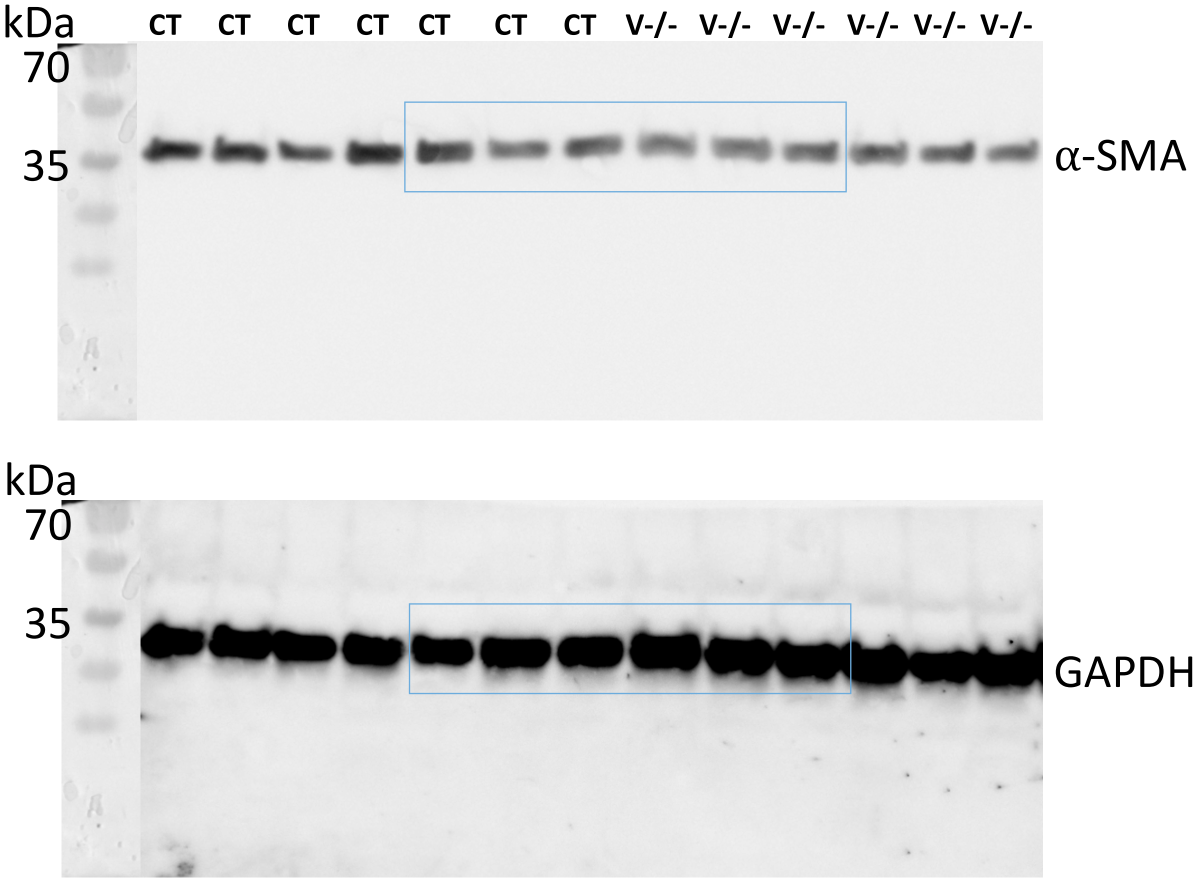

Figure 4C

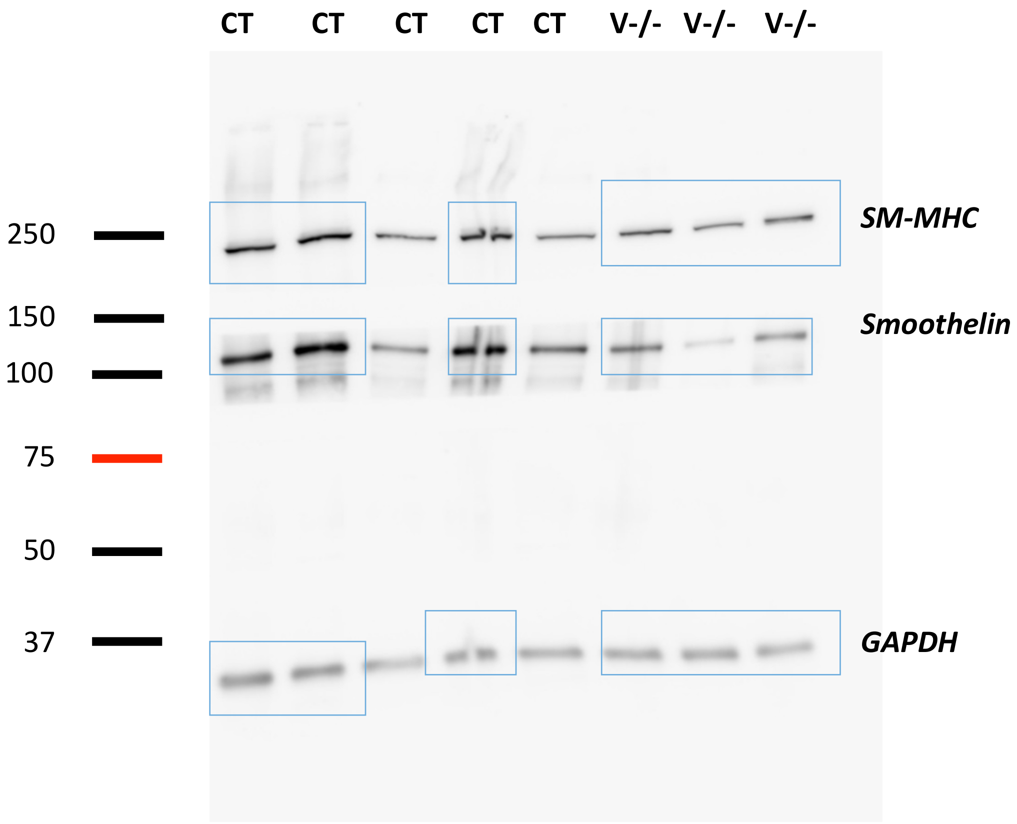

Figure 5B

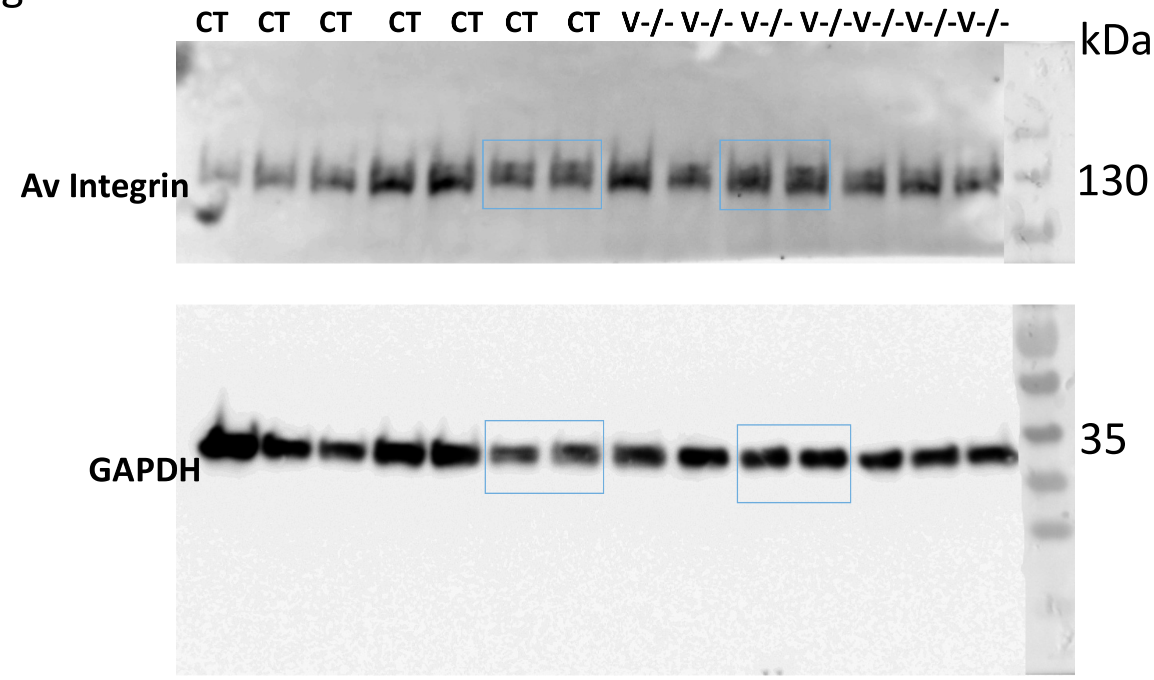

Figure 5B

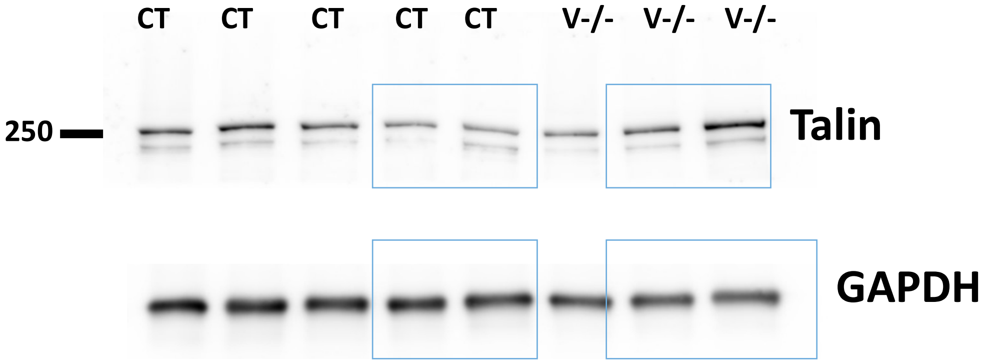

Figure 5B

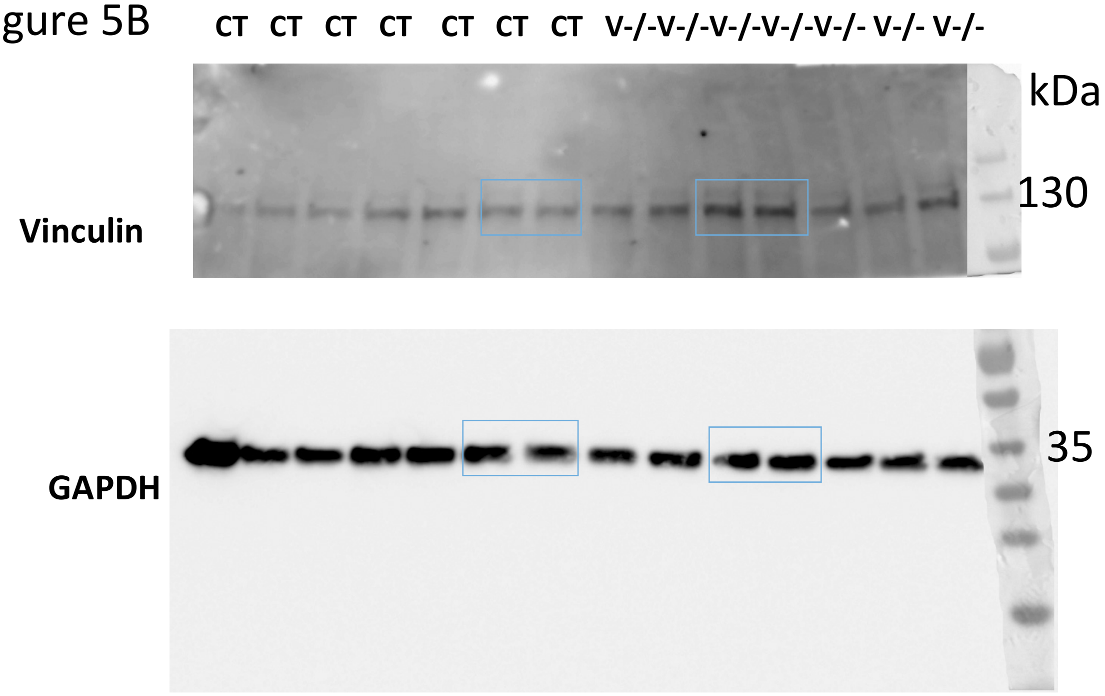

Fig 5D

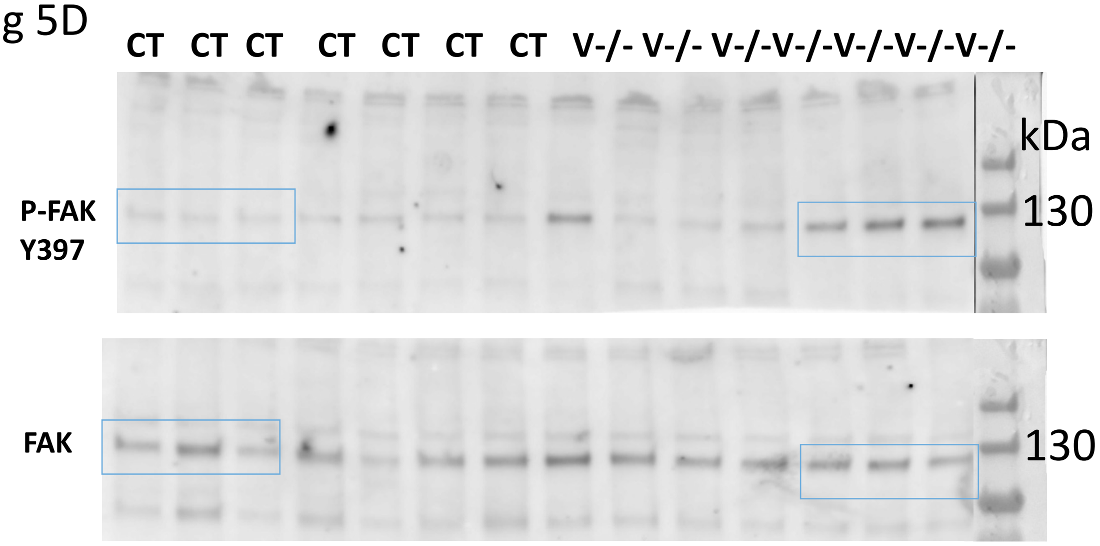

Figure 5D

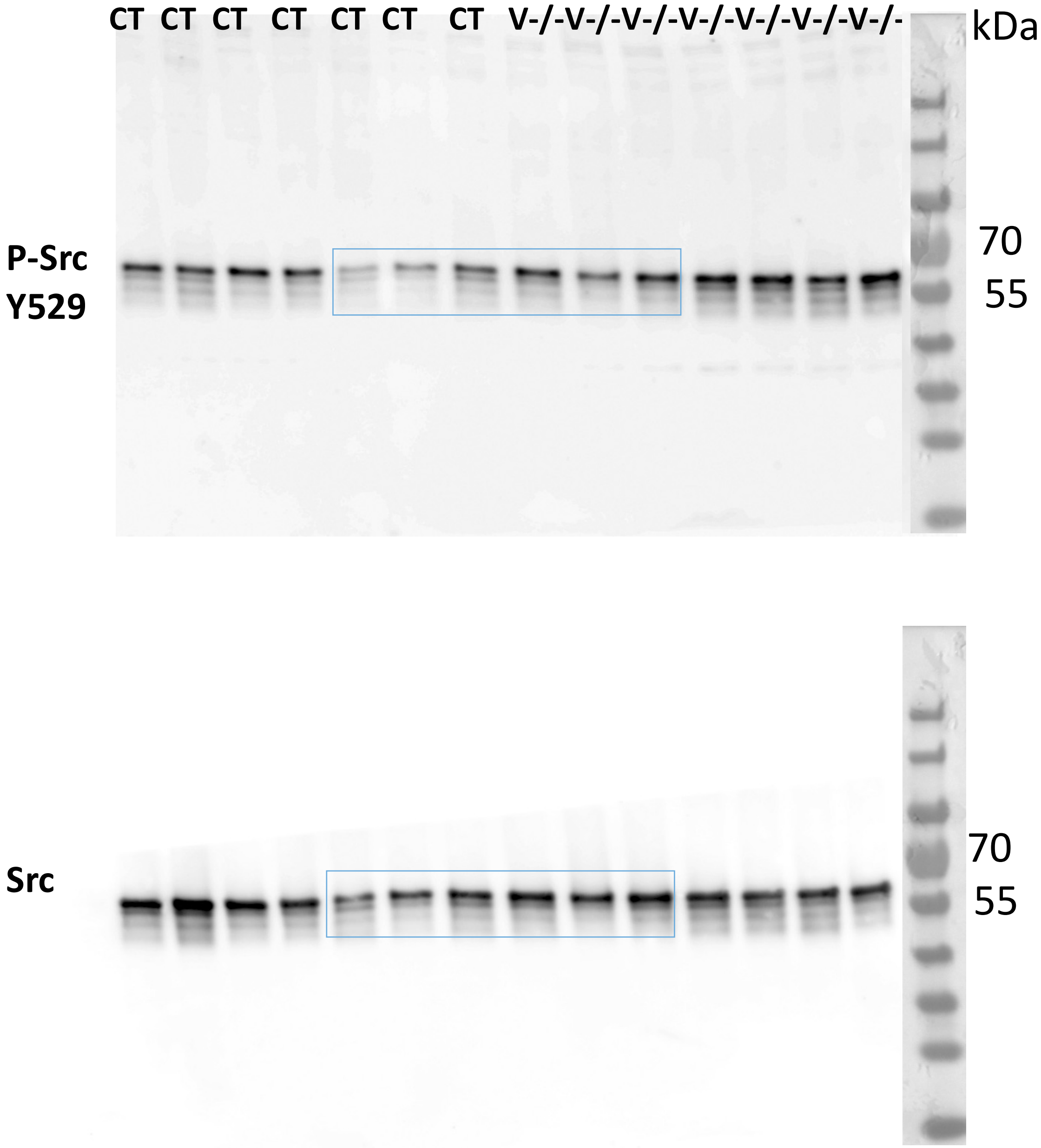

Figure 5D

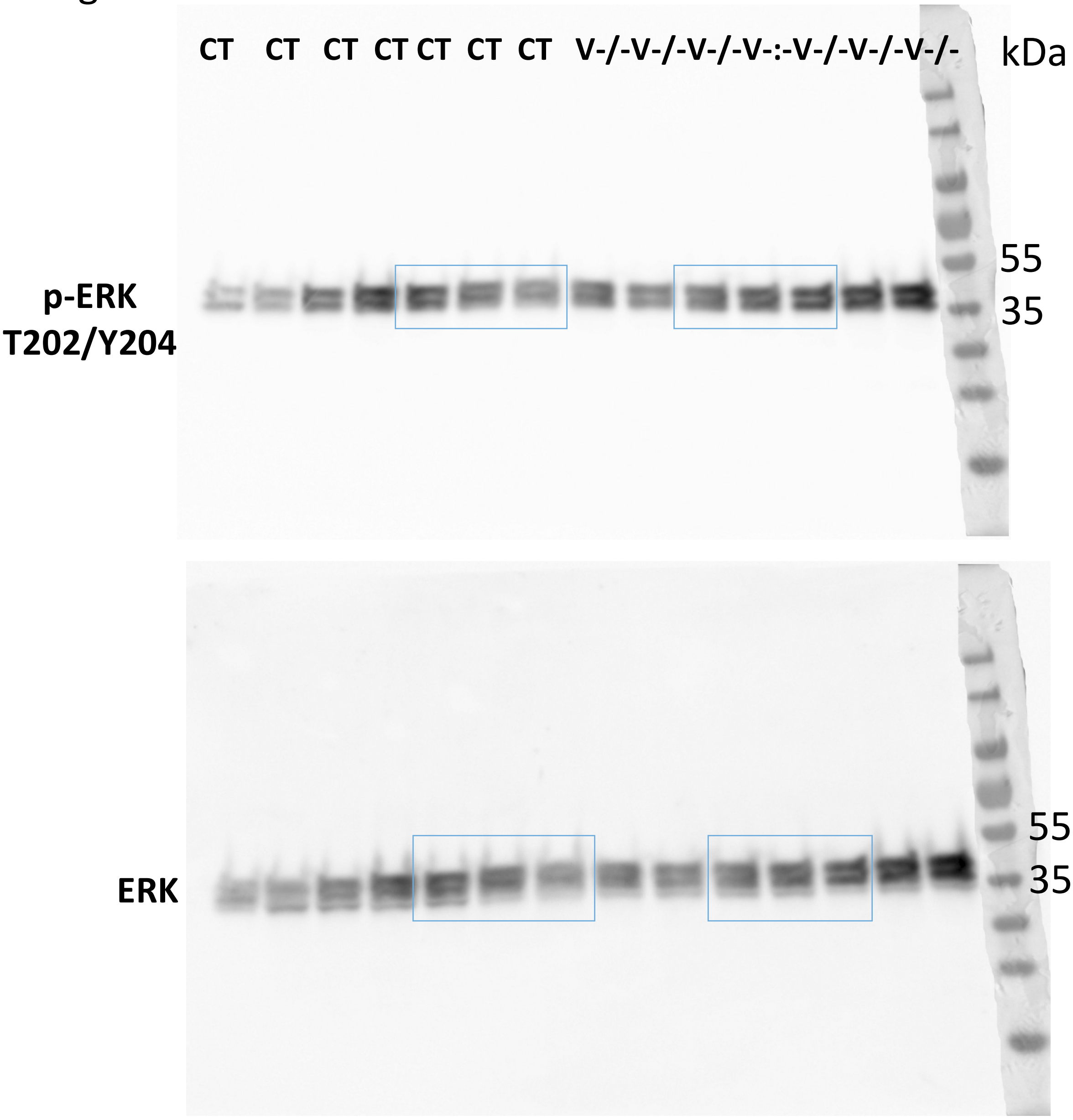

Supplementary figure S2B.

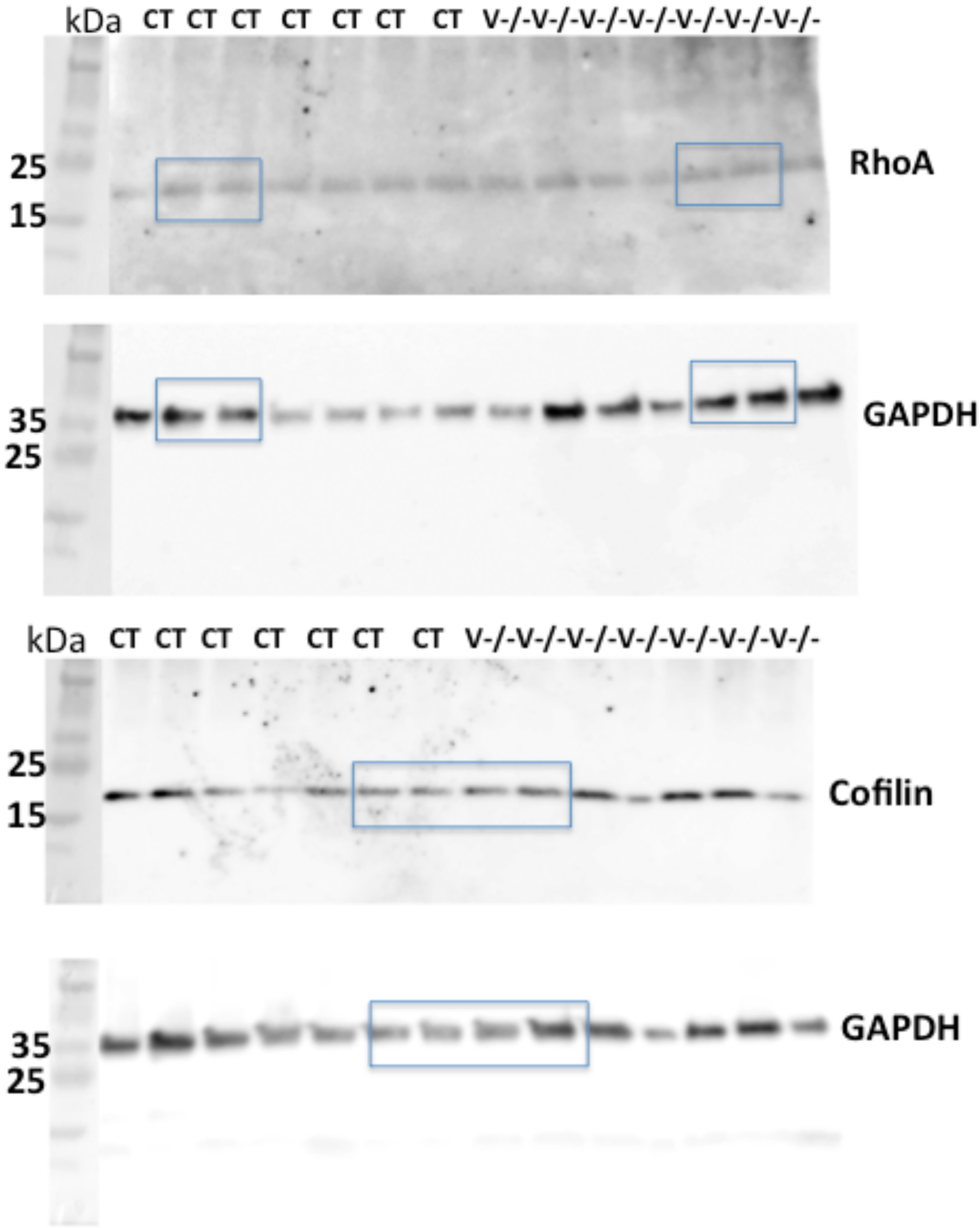

Supplementary figure S2B.

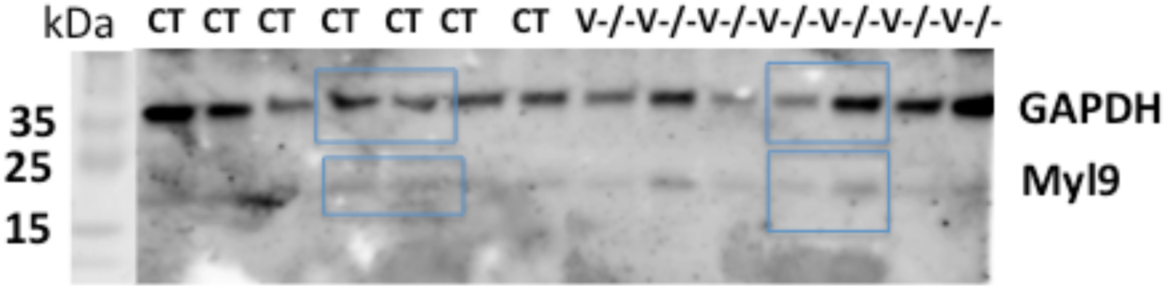

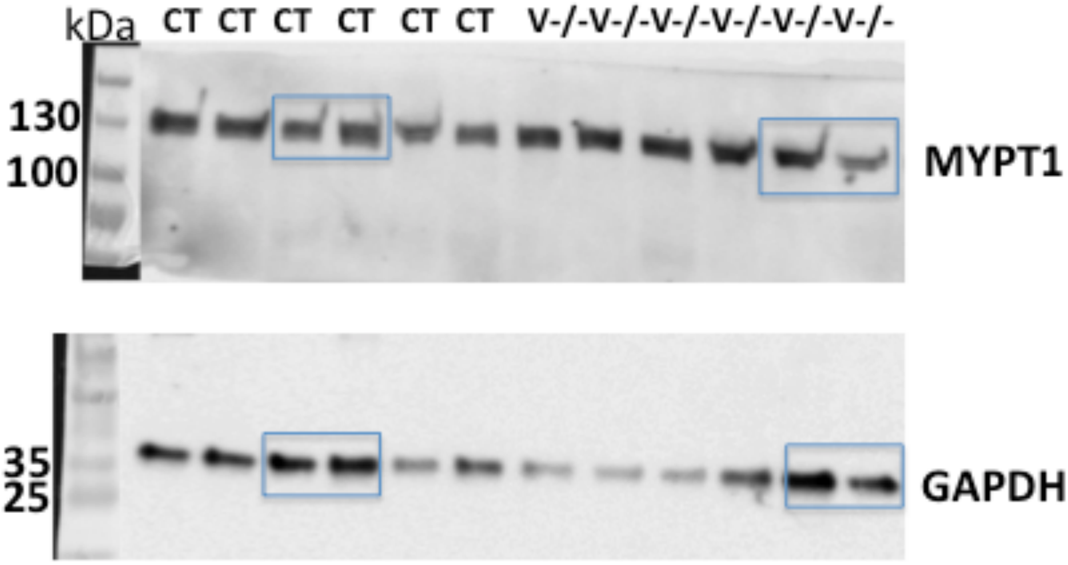

Supplementary figure S2B.

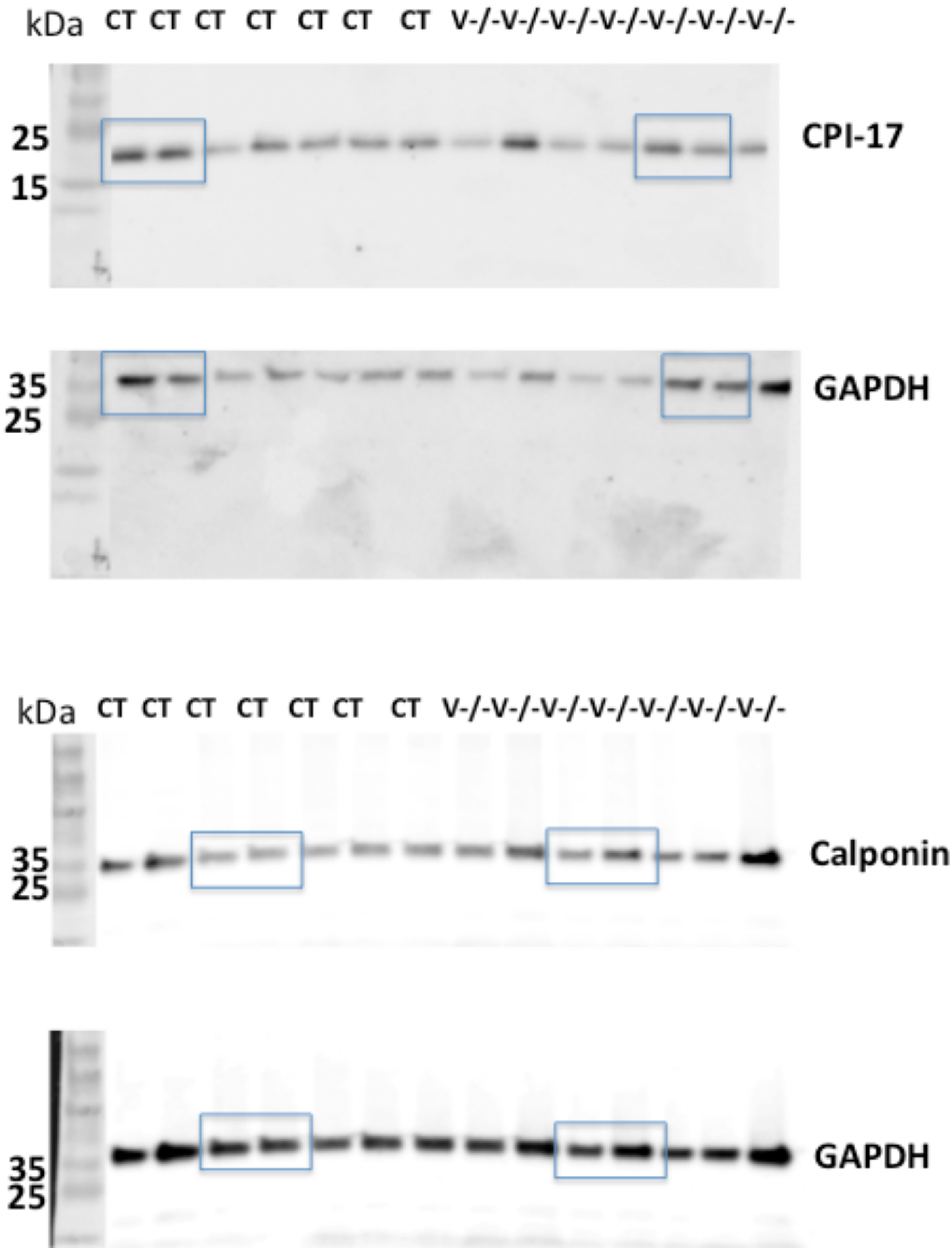

Supplementary figure S2B.

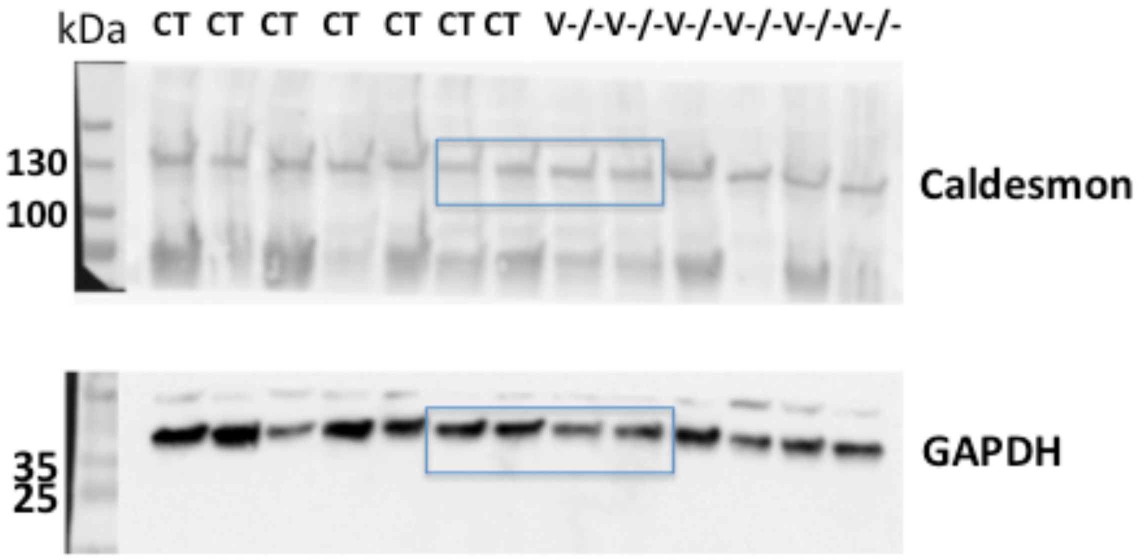

Supplement: Supplementary file 1 — Supplementary data [file 41598_2017_12024_MOESM1_ESM.pdf]
